# Enhancing precision in human neuroscience

**DOI:** 10.7554/eLife.85980

**Published:** 2023-08-09

**Authors:** Stephan Nebe, Mario Reutter, Daniel H Baker, Jens Bölte, Gregor Domes, Matthias Gamer, Anne Gärtner, Carsten Gießing, Caroline Gurr, Kirsten Hilger, Philippe Jawinski, Louisa Kulke, Alexander Lischke, Sebastian Markett, Maria Meier, Christian J Merz, Tzvetan Popov, Lara MC Puhlmann, Daniel S Quintana, Tim Schäfer, Anna-Lena Schubert, Matthias FJ Sperl, Antonia Vehlen, Tina B Lonsdorf, Gordon B Feld

**Affiliations:** 1 Zurich Center for Neuroeconomics, Department of Economics, University of Zurich Zurich Switzerland; 2 Department of Psychology, Julius-Maximilians-University Würzburg Germany; 3 Department of Psychology and York Biomedical Research Institute, University of York York United Kingdom; 4 Institute for Psychology, University of Münster, Otto-Creuzfeldt Center for Cognitive and Behavioral Neuroscience Münster Germany; 5 Department of Biological and Clinical Psychology, University of Trier Trier Germany; 6 Institute for Cognitive and Affective Neuroscience Trier Germany; 7 Faculty of Psychology, Technische Universität Dresden Dresden Germany; 8 Biological Psychology, Department of Psychology, School of Medicine and Health Sciences, Carl von Ossietzky University of Oldenburg Oldenburg Germany; 9 Department of Child and Adolescent Psychiatry, Psychosomatics and Psychotherapy, University Hospital, Goethe University Frankfurt Germany; 10 Brain Imaging Center, Goethe University Frankfurt Germany; 11 Department of Psychology, Psychological Diagnostics and Intervention, Catholic University of Eichstätt-Ingolstadt Eichstätt Germany; 12 Department of Psychology, Humboldt-Universität zu Berlin Berlin Germany; 13 Department of Developmental with Educational Psychology, University of Bremen Bremen Germany; 14 Department of Psychology, Medical School Hamburg Hamburg Germany; 15 Institute of Clinical Psychology and Psychotherapy, Medical School Hamburg Hamburg Germany; 16 Department of Psychology, University of Konstanz Konstanz Germany; 17 University Psychiatric Hospitals, Child and Adolescent Psychiatric Research Department (UPKKJ), University of Basel Basel Switzerland; 18 Department of Cognitive Psychology, Institute of Cognitive Neuroscience, Faculty of Psychology, Ruhr University Bochum Bochum Germany; 19 Department of Psychology, Methods of Plasticity Research, University of Zurich Zurich Switzerland; 20 Leibniz Institute for Resilience Research Mainz Germany; 21 Max Planck Institute for Human Cognitive and Brain Sciences Leipzig Germany; 22 NevSom, Department of Rare Disorders & Disabilities, Oslo University Hospital Oslo Norway; 23 KG Jebsen Centre for Neurodevelopmental Disorders, University of Oslo Oslo Norway; 24 Norwegian Centre for Mental Disorders Research (NORMENT), University of Oslo Oslo Norway; 25 Department of Psychology, University of Mainz Mainz Germany; 26 Department of Clinical Psychology and Psychotherapy, University of Giessen Giessen Germany; 27 Center for Mind, Brain and Behavior, Universities of Marburg and Giessen Giessen Germany; 28 Department of Systems Neuroscience, University Medical Center Hamburg-Eppendorf Hamburg Germany; 29 Department of Psychology, Biological Psychology and Cognitive Neuroscience, University of Bielefeld Bielefeld Germany; 30 Department of Clinical Psychology, Central Institute of Mental Health, Medical Faculty Mannheim, Heidelberg University Mannheim Germany; 31 Department of Psychology, Heidelberg University Heidelberg Germany; 32 Department of Addiction Behavior and Addiction Medicine, Central Institute of Mental Health, Medical Faculty Mannheim, Heidelberg University Mannheim Germany; 33 Department of Psychiatry and Psychotherapy, Central Institute of Mental Health, Medical Faculty Mannheim, Heidelberg University Mannheim Germany; National Institute of Mental Health United States; National Institute of Mental Health United States

**Keywords:** human neuroscience, precision, experimental methods, sample size, reliability, generalizability

## Abstract

Human neuroscience has always been pushing the boundary of what is measurable. During the last decade, concerns about statistical power and replicability – in science in general, but also specifically in human neuroscience – have fueled an extensive debate. One important insight from this discourse is the need for larger samples, which naturally increases statistical power. An alternative is to increase the precision of measurements, which is the focus of this review. This option is often overlooked, even though statistical power benefits from increasing precision as much as from increasing sample size. Nonetheless, precision has always been at the heart of good scientific practice in human neuroscience, with researchers relying on lab traditions or rules of thumb to ensure sufficient precision for their studies. In this review, we encourage a more systematic approach to precision. We start by introducing measurement precision and its importance for well-powered studies in human neuroscience. Then, determinants for precision in a range of neuroscientific methods (MRI, M/EEG, EDA, Eye-Tracking, and Endocrinology) are elaborated. We end by discussing how a more systematic evaluation of precision and the application of respective insights can lead to an increase in reproducibility in human neuroscience.

## Introduction

Understanding the functional organization of the human mind depends on the type, quality, and particularly the precision of the measurements employed in research. Experimental research in human neuroscience involves multiple steps (designing and conducting a study, data processing, statistical analyses, reporting results) – each involving many parameters and decisions between (often) equally valid options. This so-called ‘garden of forking paths’ during the research process has received considerable attention (Gelman and Loken, 2014), as it has been demonstrated that findings critically depend on design, processing, and analysis pipelines (Botvinik-Nezer et al., 2020; Carp, 2012). Analytical heterogeneity can have a crucial impact on measurement precision (Moriarity and Alloy, 2021) and consequently on statistical power and sample size requirements (Button et al., 2013). In this review, we focus on the often-neglected question of how to optimize measurement precision in human neuroscience and discuss implications for power analyses. Knowledge about these factors will strongly benefit neuroscientists interested in individual differences, group-level effects, and biomarkers for disorders alike as different research questions profit from different optimization strategies. Many of these factors are passed on by lab traditions but are not necessarily well documented in the published literature or evaluated empirically. For example, factors such as the number of trials per condition, tolerance for sensor noise, scanner pulse sequences, and electrode positions are often based on previous work in a given lab rather than a solid quantitative principle. Therefore, there is an urgent need to synthesize the available empirical evidence on the determinants of precision and to make this knowledge available to the neuroscience research community, which requires the sharing of original data using standardized reporting formats (e.g., BIDS, https://bids.neuroimaging.io/specification.html).

**Figure 1.**
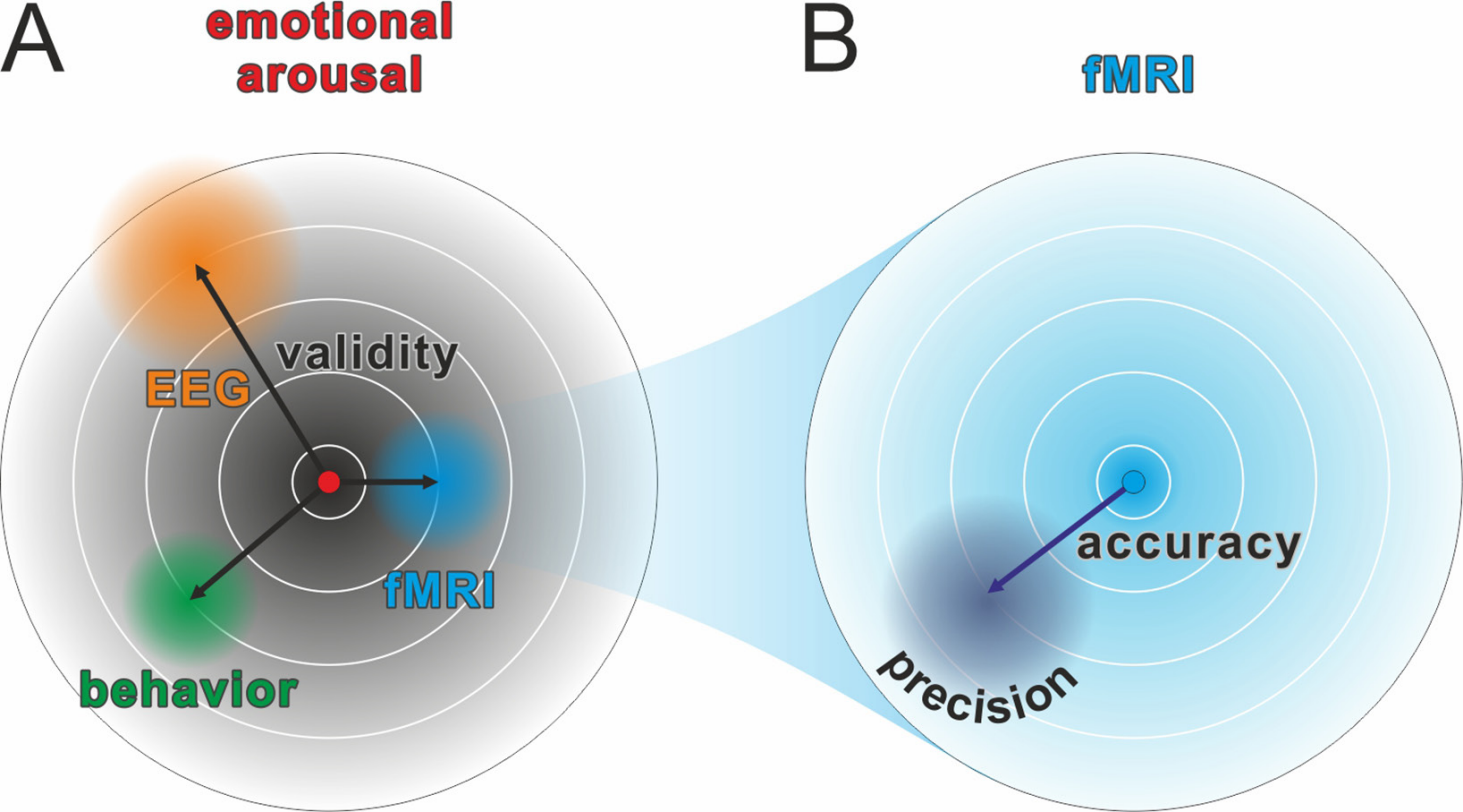
Comparison of validity, precision, and accuracy. (**A**) A latent construct such as emotional arousal (red dot in the center of the circle) can be operationalized using a variety of methods (e.g., EEG ERN amplitudes, fMRI amygdala activation, or self-reports such as the Self-Assessment Manikin). These methods may differ in their construct validity (black arrows), that is, the measurement may be biased away from the true value of the construct. Of note, in this model, the true values are those of an unknown latent construct and thus validity will always be at least partially a philosophical question. Some may, for example, argue that measuring neural activity directly with sufficient precision is equivalent to measuring the latent construct. However, we prescribe to an emergent materialism and focus on measurement precision. The important and complex question of validity is thus beyond the scope of this review and should be discussed elsewhere. (**B**) Accuracy and precision are related to validity with the important difference that they are fully addressed within the framework of the manifest variable used to operationalize the latent construct (e.g., fMRI amygdala activation). The true value is shown as a blue dot in the center of the circle and, in this example, would be the true activity of the amygdala. The lack of accuracy (dark blue arrow) is determined by the tendency of the measured values to be biased away from this true value, that is, when signal losses to deeper structures alter the blood oxygen-level dependent (BOLD) signal measuring amygdala activity. Oftentimes, accuracy is unknown and can only be statistically estimated (see Eye-Tracking section for an exception). The precision is determined by the amount of error variance (diffuse dark blue area), i.e. precision is high if BOLD signals measured at the amygdala are similar to each other under the assumption that everything else remains equal. The main aim of this review is to discuss how precision can be optimized in human neuroscience.

**Figure 2.**
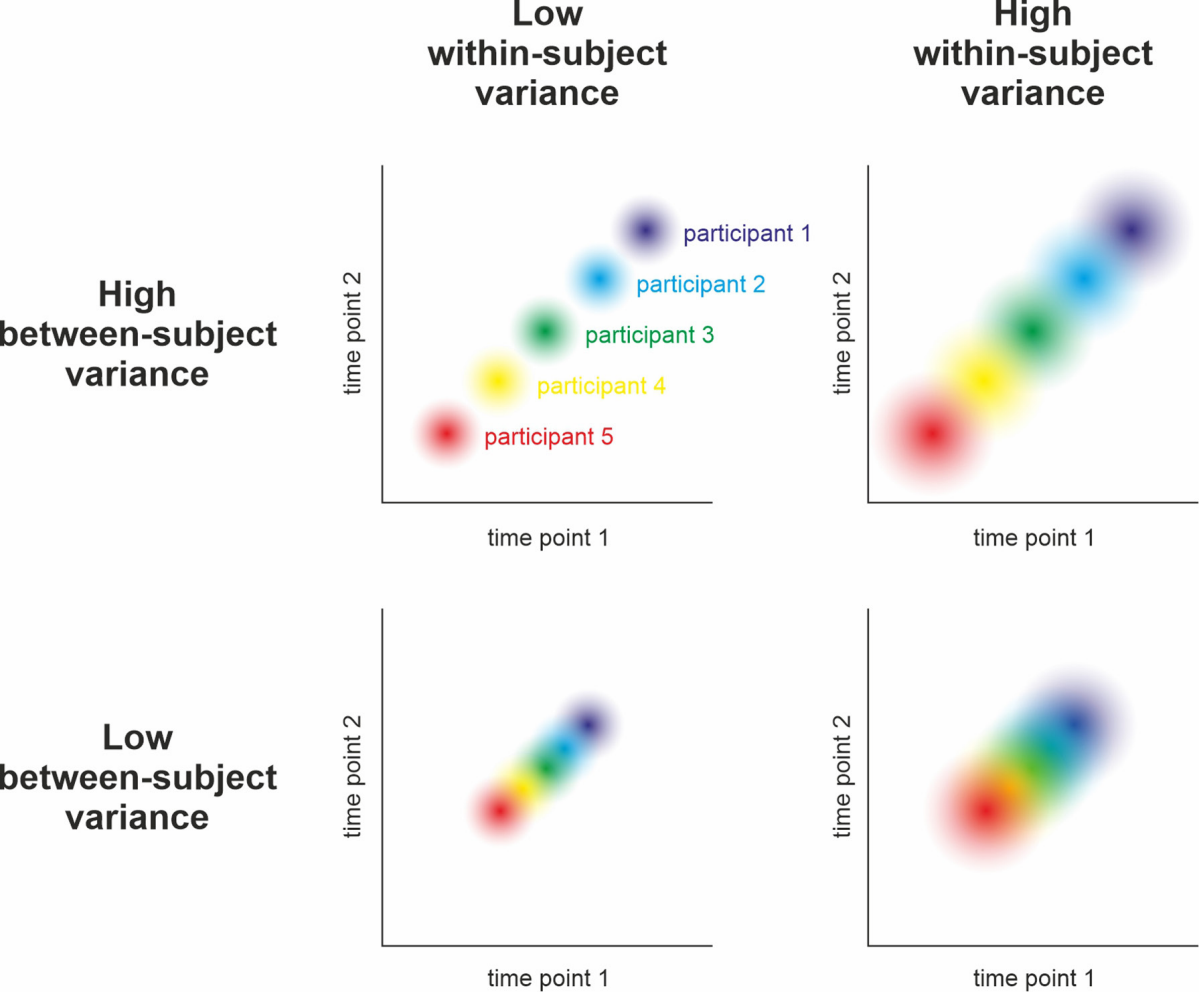
Relation between reliability and precision. Hypothetical measurement of a variable at two time points in five participants under different assumptions of between-subjects and within-subject variance. Reliability can be understood as the relative stability of individual *z*-scores across repeated measurements of the same sample: Do participants who score high during the first assessment also score high in the second (compared to the rest of the sample)? Statistically, its calculation relies on relating the within-subject variance (illustrated by dot size) to the between-subjects variance (i.e., the spread of dots). As can be seen above and in , high reliability is achieved when the within-subject variance is small and the between-subjects variance is large (i.e., no overlap of dots in the top left panel). Low reliability can occur due to high within-subject variance and low between-subjects variance (i.e., highly overlapping dots in the bottom right) and intermediate reliability might result from similar between- and within-subject variance (top right and bottom left). Consequently, reliability can only be interpreted with respect to subject-level precision when taking the observed population variance (i.e., the group-level precision) into account (see ). For example, an event-related potential in the EEG may be sufficiently reliable after having collected 50 trials in a sample drawn from a population of young healthy adults. The same measure, however, may be unreliable in elderly populations or patients due to increased within-subject variance (i.e., decreased subject-level precision). Figure 2—source code 1.Reliability, between & within variance.This R code simulates four samples of 50 subjects with 50 trials each using different degrees of variability within and between subjects. The results indicate that (odd-even) reliability is best at a combination of low within-subject variance but high between-subjects variability (leading to lower group-level precision). Reliability declines when within- and between-subjects variance are both high or low and is worst when the former is high but the latter is low. The results support the claims illustrated in Figure 2. Figure 2—source code 2.Reliability & (between) SD.This R code simulates two samples of 64 subjects with 100 trials each using vastly different degrees of between-subjects variance (but constant within-subject variability, leading to constant subject-level precision). The results show that, everything else being equal, homogenous samples (i.e., with low between-subjects variance) optimize group-level precision at the expense of reliability (Hedge et al., 2018), while heterogenous samples optimize reliability at the expense of group-level precision.

We define measurement precision as the ability to repeatedly measure a variable with a constant true score and obtain similar results (Cumming, 2014). Therefore, precision will be highest if the measurement is not affected by noise, measurement errors, or uncontrolled covariates. Crucially, precision is related to yet distinct from other concepts such as validity, accuracy, or reliability (see Figures 1 and 2 for the relation of precision to other concepts and the Glossary in the Appendix for explanations of the most important terms). The higher the precision on a participant- or group-level, the higher the statistical power for detecting effects across participants or between groups of participants, respectively. Thus, a more precise measurement increases the probability of detecting a true effect. Additionally, this results in a more accurate estimation of effect sizes that can be used for future power calculations. Research projects that are based on proper power calculations help to produce less ambiguous results and, ultimately, lead to a more efficient use of research funds.

Although high measurement precision is a key determinant of statistical power, it has often been neglected. Rather, increasing sample size has evolved as the primary approach to augmenting statistical power in psychology (Open Science Collaboration, 2015) and neuroscience (Button et al., 2013). Generally, statistical power of a study on group differences is determined by the following parameters: (a) the chosen threshold of statistical significance α, (b) the unstandardized effect size relative to the total variance, and (c) the total sample size *N*. This model can be converted to obtain the total sample size needed to achieve a desired statistical power for simple statistical analyses (e.g., the main effect of an ANOVA), given an expected effect size (e.g., *f* for ANOVA models) and significance level (Button et al., 2013; G*Power Faul et al., 2009). Numerous researchers have previously called for increased sample sizes in human neuroscience to achieve adequate statistical power (e.g., Button et al., 2013; Szucs and Ioannidis, 2020). However, the cost of acquiring neuroscience data is comparatively high, considering preparation time, consumables, equipment operating costs, staff training, and financial compensation for participants. External resource constraints often render the results of a priori power analyses meaningless if the number of participants cannot be easily increased (Lakens, 2022).

Raising the total sample size is only one possible way to increase statistical power. A promising alternative is to enhance precision at the aggregation level of interest. This can be achieved on group-level by an adequate selection of the sample and/or paradigm (Hedge et al., 2018), on the subject-level by increasing the number of trials (Baker et al., 2021; Boudewyn et al., 2018; Chaumon et al., 2021), or even on the trial-level by using more precise measurement techniques. Conversely, a lack of measurement precision results in an increased amount of error variance and, thus, increases the estimate of total variance. Critically, determining the gain in precision from increasing the trial count is not trivial. While extending the number of participants provides independent observations and predictable merit, additional trials can increase the impact of sequence effects (e.g., habituation, fatigue, or learning). Consequently, increasing the number of trials will not indefinitely benefit measurement precision and reliability (see Figure 2 for a delimitation of both terms), although sequence effects can be mitigated by including breaks or by modeling them (e.g., Sperl et al., 2021).

Measurement precision is beneficial for statistical power in multiple ways. In the following, we compiled a summary of these factors in the context of different biopsychological and neuroscientific methods. We provide information on their possible influence on measurement precision (and related concepts, see Glossary in Appendix) and describe future avenues to quantifying influences of under-researched variables that may affect measurement precision to an unknown degree. We encourage neuroscientists to comprehensively assess and report these determinants in the future, and also to consolidate empirical evidence about the magnitude of their impact on measurement precision. Furthermore, we motivate basic research on this topic to identify conditions in which the influence of certain factors may be particularly important or negligible.

## Measurement-specific considerations

In the following section, we focus on five different neuroscientific and psychophysiological methods to exemplify different aspects related to precision: We begin with magnetic resonance imaging (MRI) to illustrate how the utilization of covariates can reduce error variance. Subsequently, we focus on magneto- and electroencephalography (M/EEG) to explain how aggregation across repeated measures is another option to reduce unsystematic noise. Next is electrodermal activity, which provides a prime example of a change in the signal of interest due to sequence effects (especially habituation). Afterwards, eye-tracking is used to illuminate the interplay of precision and accuracy (Figure 1B). Finally, the impact of biological rhythms on hormone expression is demonstrated in the section on endocrinology. Vitally, the concepts exemplified in each subsection are not specific to the presented neuroscientific method and should thus be considered for every neuroscientific study (more comprehensive information can be found in the Table of Resources in Supplementary file 1). We conclude these sections of the manuscript by identifying seven issues that should be considered to ensure adequate precision when collecting multiple neuroscience measures simultaneously.

### Magnetic resonance imaging (MRI)

Functional MRI (fMRI) is an indirect measure of brain activity, which captures the change in flow of oxygenated blood. Structural MRI creates images of brain tissues, allowing anatomical studies as well as estimation of the distribution of cell populations or connections between brain regions.

#### Design and data recording

The most important property of an MRI scanner is its field strength. Typical values are 1.5, 3, or 7 Tesla, with higher values leading to improved spatial resolution due to increased signal-to-noise ratios but increasing the likelihood of side effects for participants as well as artifacts (Bernstein et al., 2006; Polimeni et al., 2018; Theysohn et al., 2014; Uğurbil, 2018). Furthermore, parameters of the scan protocol impact *what* is measured. For instance, the field of view can be adapted to achieve best precision in specific brain regions, or the repetition time can be adjusted to focus on temporal versus spatial precision (Mezrich, 1995). Moreover, strategies to reduce movement (e.g., increasing temporal resolution and thereby potentially reducing acquisition time through multi-band sequences, fixating the head with cushions, training in a mock scanner, real-time feedback) (Horien et al., 2020; Risk et al., 2018) and modeling physiological noise (e.g., heartbeat and breathing) can reduce error variance in analyses of BOLD signals and thus increase precision. Finally, a larger number of trials per subject in task-based fMRI studies or a longer duration of scanning in resting-state studies increases the precision of the signal (Baker et al., 2021; Gordon et al., 2017; Noble et al., 2017). However, longer scanning durations may lead to effects of fatigue or reduced motivation in subjects, which can be counteracted by dividing the data acquisition into several shorter scanning blocks (Laumann et al., 2015).

##### Functional magnetic resonance imaging: Studying brain activation

fMRI measures neural activity indirectly by assessing electromagnetic properties of local blood flow. Several factors at the subject- and group-level affect precision, including design efficiency and factors reducing error variance (Mechelli et al., 2003). Design efficiency reflects whether the contrasted trials induce a large variability in signal change and, therefore, improves signal-to-noise ratio. To increase it, we can, for example, ‘jitter’ inter-stimulus intervals (i.e., adding a random duration to each inter-stimulus interval), include null events (i.e., trials with the same timing and duration than other trials in the experiment but without presenting any sensory input different from the inter-trial interval to the participants), or optimize the order of trials (Friston et al., 1999; Kao et al., 2009; Wager and Nichols, 2003). Block designs, in which one experimental condition is presented several times in succession, often have greater design efficiency than event-related designs, in which condition blocks are presented in randomized order. However, block designs may introduce sequence effects (e.g., expectation and context effects) that can increase error variance, reducing precision (Howseman et al., 1999). In addition, multi-band acquisition of fMRI can increase the temporal resolution greatly and, thus, increases the amount of data per trial per subject. However, multi-band fMRI might decrease the signal-to-noise ratio (Todd et al., 2017) and was found to compromise detection of reward-related striatal and medial prefrontal cortical activation (Srirangarajan et al., 2021). In turn, multi-echo imaging in combination with adequate denoising techniques can increase the precision in fMRI in general (Lynch et al., 2021) and can even counter the detrimental effects of multi-band imaging on precision (Fazal et al., 2023). Lastly, the temporal frequencies of the experimental signal should match the optimal filter characteristics of the hemodynamic response function (~0.4 Hz) and not strongly overlap with low-frequency components, which are often considered as noise and filtered out in the following analysis (Della-Maggiore et al., 2002).

##### Connectivity and brain networks

Brain connectivity can be assessed on a functional or structural level. For structural connectivity, measurement precision depends on a large number of acquired diffusion weighted images. However, methods have been proposed to achieve good precision even with small amounts of data (Zheng et al., 2021; Wehrheim et al., 2022). With respect to functional resting-state connectivity, there is a debate about comparing fMRI data of varying lengths and the loss in precision when using insufficient scanning durations (Airan et al., 2016; Gordon et al., 2017; Miranda-Dominguez et al., 2014). As resting-state scans are unconstrained states by definition, other factors also influence the precision of the measurement, for example, whether participants have their eyes open or closed (Patriat et al., 2013).

### Data analysis

#### Preprocessing

There are various software tools for analyzing MRI data, for example, FSL (Jenkinson et al., 2012), SPM (Ashburner, 2012), FreeSurfer (Fischl, 2012), and AFNI/SUMA (Saad et al., 2004). All analyses require data pre-processing, for which different pipelines have been proposed with regard to both structural (Clarkson et al., 2011) and functional analyses. These pipelines differ, for example, in the quality of normalization of individual brains into a standard space or motion correction (Esteban et al., 2019; Strother, 2006). An essential step to improve precision is to apply thorough quality assessment (QA) methods to the pre-processed data. For structural data, the manual ENIGMA QA protocol (ENIGMA, 2017) or automated quality metrics (Esteban et al., 2017) have been shown to improve data quality (Chow and Paramesran, 2016).

#### General approach

**Figure 3.**
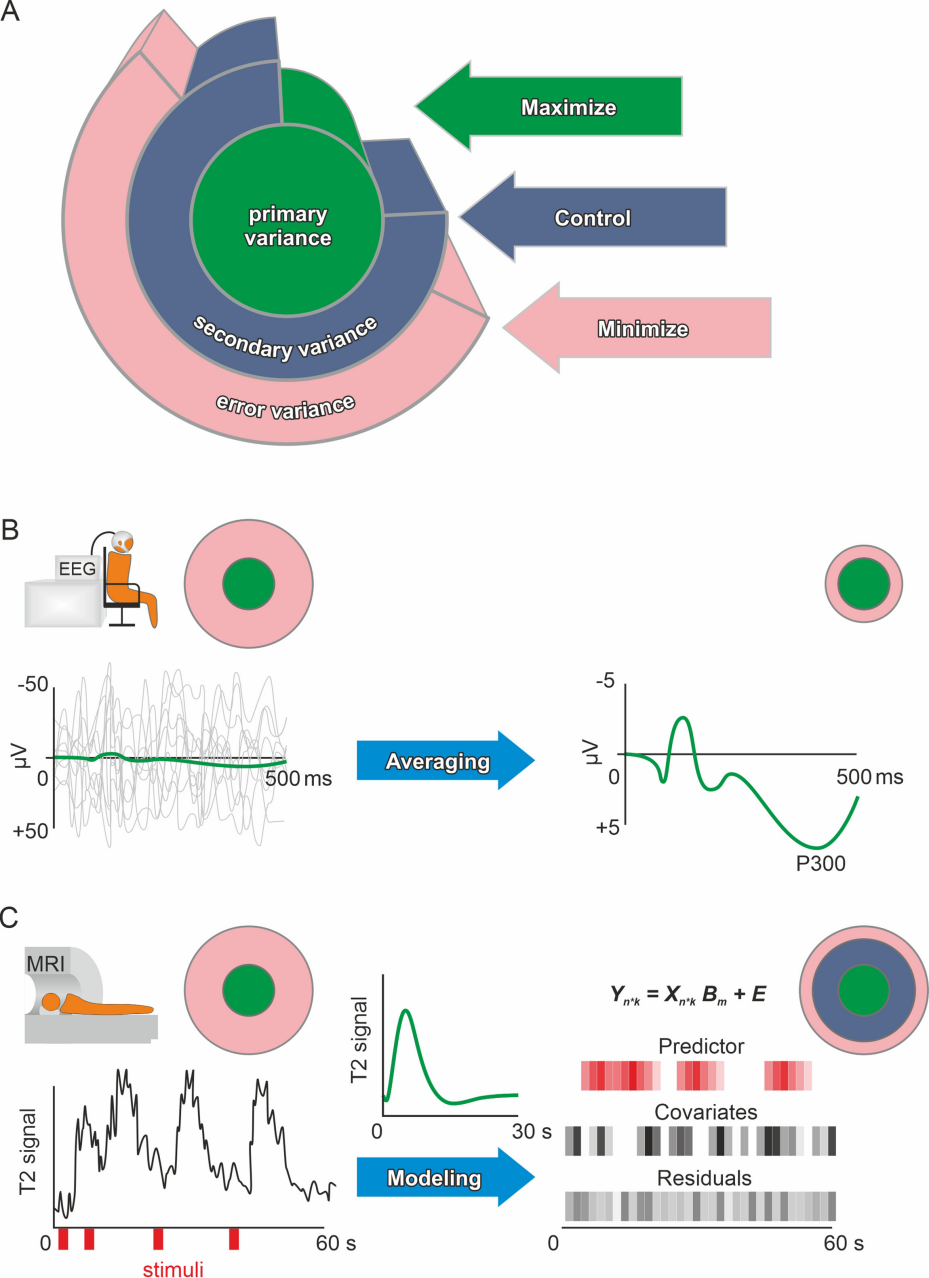
Primary, secondary, and error variance. (**A**) There are three main sources of variance in a measurement, each providing a different angle on optimizing precision. Primary (or systematic) variance results from changes in the true value of the manifest (dependent) variable upon manipulation of the independent variable and therefore represents what we desire to measure (e.g., neuronal activity due to emotional stimuli). Secondary variance is attributable to other variables that are not the focus of the research but are under the experimenter’s control, for example, the influence of the menstrual cycle on neural activity can either be controlled by measuring all participants at the same time of the cycle or by adding time of cycle as a covariate to the analysis. Trivially, if the research topic was the effect of the menstrual cycle on neural activity, then this variance would be primary variance, highlighting that these definitions depend solely on the research question. Error variance is any change in the measurement that cannot be reasonably accounted for by other variables. It is thus assumed to be a random error (see systematic error for exceptions). Explained variance (see definition of effect size in the Glossary in Appendix) is the size of the effect of manipulating the independent variable compared to the total variance after accounting for the measured secondary variance (via covariates). Precision is enhanced if the error variance is minimized and/or the secondary variance is controlled. Methods in human neuroscience differ substantially in the way they deal with error variance. (Kerlinger, 1964, for the first description of the Max-Con-Min principle). (**B**) In EEG research, a popular method is averaging. On the left, the evoked neuronal response (primary variance – green line) of an auditory stimulus is much smaller than the ongoing neuronal activity (error variance – gray lines). Error variance is assumed to be random and, thus, should cancel out during averaging. The more trials (many gray lines on the left) are averaged, the less error variance remains if we assume that the underlying true evoked neuronal response remains constant (green subject-level evoked potential on the right). Filtering and independent component analysis are further popular methods to reduce error variance in EEG research. After applying these procedures on the subject-level, the data can be used for group-level analyses. (**C**) In fMRI research, a linear model is commonly used to prepare the subject-level data before group analyses. The time series data are modeled using beta weights, a design matrix, and the residuals (see GLM and mass univariate approaches in the Glossary in Appendix). Essentially, a hypothetical hemodynamic response (green line in the middle) is convolved with the stimuli (red) to form predicted values. Covariates such as movements or physiological parameters are added. Therefore, the error variance (residuals) that remains is the part of the time series that cannot be explained by primary variance (predictor) or secondary variance (covariates). Of course, averaging and modeling approaches can both be used for the same method depending on the researcher’s preferences. Additionally, pre-processing procedures such as artifact rejection are used ubiquitously to reduce error variance.

For the analysis of MRI data, a general linear model (GLM, Friston et al., 1999) is commonly used in a mass-univariate approach (see Figure 3C). Here, precision mainly depends on the data quality and sample composition. Moreover, error variance can be reduced by adding covariates (e.g., participant movement for functional analyses; and age, sex/gender, handedness, and total intracranial volume for structural analyses). Furthermore, physiological noise from heartbeat or breathing can be modeled and, thus, corresponding noise decreased (Chang et al., 2009; Havsteen et al., 2017; Kasper et al., 2017; Lund et al., 2006). Note, however, that the univariate analysis approach has been shown to have inferior retest-reliability compared to multivariate analyses (Elliott et al., 2020; Kragel et al., 2018). For this reason, some researchers generally recommended multivariate over univariate analyses (Kragel et al., 2021; Noble et al., 2019). In addition, the intake of all substances that interact with central nervous activity or blood flow in the brain should be assessed. These are likely to have an effect on fMRI, but there are no general guidelines on how to deal with them. While excluding participants who regularly consume nicotine, alcohol, or caffeine would greatly reduce the generalizability, not accounting for different exposures to these psychoactive substances increases error variance and, thus, reduces measurement precision. Therefore, the level of regular consumption and the time since last intake could be assessed and used as covariate to control for systematic variation in BOLD responses due to the effects of the substance.

#### Functional magnetic resonance imaging: Studying brain activation

Functional magnetic resonance imaging data are usually analyzed using a two-level summary approach. First-level models analyze the individual subject’s BOLD time series and estimate summary statistics (such as individual contrast-weighted GLM coefficients, see Figure 3) that are further investigated at the second or group-level (Penny and Holmes, 2004). At the group-level, estimated effects depend on the precision of the subject-level estimations, also benefiting from the previously mentioned use of covariates and random effects (Penny and Holmes, 2004). Furthermore, one can model serial autocorrelation and deviations from the canonical hemodynamic response function, and apply frequency filters that preserve the experimentally induced BOLD signal but reduce error-related signals in first-level analyses (Friston et al., 2007).

In contrast to voxel-wise univariate analyses, multivariate analysis approaches combine information across voxels, for example, to distinguish different groups or to predict behavior (Haxby et al., 2014). Some of these approaches account for large parts of the variance in the predictor space (principal component regression) or in both the predictor and outcome space (partial least squares, Frank and Friedman, 1993). Regularized regression approaches such as elastic nets, LASSO (Least Absolute Shrinkage and Selection Operator) analyses, or ridge regression can serve the same purpose by incorporating information of only few or many voxels (Gießing et al., 2020).

#### Connectivity and brain networks

The analysis step of parcellation assigns each voxel of the acquired neural data into separate regions of the brain, which are then used as nodes in the network, between which the connections (edges) are estimated. Various parcellation schemes using different criteria such as anatomical landmarks, cytoarchitectonic boundaries, fiber tracts, or functional coactivations to define those network nodes were developed and used in previous research (López-López et al., 2020; Passingham et al., 2002; Schleicher et al., 1999). For the construction of functional brain networks, functional parcellation schemes are often used assigning voxels according to their coactivations (e.g., the Local-Global Schaefer 200, Schaefer et al., 2018) or multimodal templates with consistent boundaries across different modalities (Glasser et al., 2016). In some cases, the original parcellation schemata include only cortical regions and have later been extended to subcortical brain areas (López-López et al., 2020). The choice of the optimal parcellation depends on the specific research question and results should ideally be replicated with different parcellations (Arslan et al., 2018; Bryce et al., 2021). Furthermore, current evidence suggests that analyses of time-resolved functional connectivity might profit from templates developed on patterns of dynamic functional connectivity (Fan et al., 2021).

Thus, precise parcellation is the basis to ensure meaningful connectivity patterns (Zalesky et al., 2010) and using a standard atlas for parcellation facilitates meta-analytic work and increases comparability across various studies. However, previous studies have also shown that functional parcellations of the brain vary from person to person as well as over time (Kong et al., 2019). The use of an individual parcellation template created for each subject at a specific time point separately can improve the prediction of behavioral performance, provided that the individual templates are calculated based on fMRI datasets of sufficient long scanning duration (Gordon et al., 2017; Kong et al., 2021). Another important aspect specific for task-related connectivity is the removal of task-evoked brain activation, which can be achieved by basis set task regression (e.g., Cole et al., 2019). If functional brain networks are analyzed as graphs, global metrics instead of node-specific measures have higher precision (Braun et al., 2012). There are also recommendations for dynamic connectivity analyses (Lurie et al., 2020). The highest temporal precision can be achieved by temporally resolving the correlation metric itself. Such analyses might even allow network construction of every single sample point (Faskowitz et al., 2020; Zamani Esfahlani et al., 2020). Functional brain networks have further been used as input for machine learning-based models to increase measurement precision by ‘learning’ the most relevant features of connectivity (Cwiek et al., 2022; Nielsen et al., 2020).

Concerning measurement precision of structural connectivity analyses, the use of parcellation atlases based on anatomical similarities like the Desikan-Killiany atlas (Desikan et al., 2006) or the Destrieux parcellation (Destrieux et al., 2010) is recommended (Pijnenburg et al., 2021). Multimodal atlases like the HCP Glasser parcellation (Glasser et al., 2016) are preferable when structural and functional connectivity are estimated simultaneously (Damoiseaux and Greicius, 2009; Rykhlevskaia et al., 2008). Structural connections can be modeled based on probabilistic or deterministic tractography and both methods have advantages, while multi-fiber deterministic tractography (or properly thresholded probabilistic tractography) evolved as the best solution (Sarwar et al., 2019). However, even with the gold standard analysis techniques, issues remain if fibers cross within one voxel (Jones et al., 2013; Schilling et al., 2018; Seunarine and Alexander, 2009) or when multiple fibers converge in one voxel and run in parallel before separating again (Schilling et al., 2022) resulting in reduced precision of connectivity estimates. Several approaches for data acquisition or analysis have been suggested to address these issues (Landman et al., 2012; Sedlar et al., 2021). Other issues concern the use of symmetric (recommended) versus asymmetric connectivity matrices, or the correction for node size (as discussed in Yeh et al., 2021).

### Reporting standards

For fMRI studies, previous work has established reporting standards (Nichols et al., 2017; Poldrack et al., 2008; eCOBIDAS, https://osf.io/anvqy/) as well as a standardized data structure (BIDS, Gorgolewski et al., 2016; see also Table of Resources in Supplementary file 1). Furthermore, a recently published pre-registration template provides an exhaustive list of information related to fMRI studies, which might be considered not only during pre-registration but also when reporting a completed study (Beyer et al., 2021).

### Magneto- and electroencephalography (M/EEG)

Postsynaptic currents within neural collectives generate an electro-magnetic signal that can be measured at the scalp surface by magneto- and electroencephalography (M/EEG). Signal quality depends substantially on the sensor technology (for detailed guidelines, see Gross et al., 2013; Keil et al., 2014). Gel-based EEG systems provide excellent signal quality but take time to apply. Newer dry-electrode systems are noisier but offer near-instantaneous set up. Systems using a sensor net and saline solution are a middle ground. Signal fidelity can be improved by using active electrode systems that amplify signals at the sensor or by systems with inbuilt electrical shielding. The choice of sensor technology trades off against other constraints, for example a system with fast set-up time may be desired when testing infants. In traditional cryogenic MEG systems, the sensors are fixed in a helmet, meaning that the distance from the participant’s head may vary substantially, which can affect signal strength (Stolk et al., 2013). Newer sensor technology is based on optically pumped magnetometers that avoid this issue (Hill et al., 2020).

#### Design and data recording

When designing M/EEG experiments, the trial number and sample size play an important role. Currently, the average sample size per group for M/EEG experiments is as low as 21 (Clayson et al., 2019), while large-scale replication attempts, such as EEGManyLabs (Pavlov et al., 2021), aim to test larger samples. Preparation by well-trained operators ensures similar preparation time, consistent positioning in the dewar (MEG), and comparable and reasonable impedances (EEG) across participants. Impedances (Kappenman and Luck, 2010) may differ across the scalp, depending on various factors (e.g., skull thickness, hair, hair products, and age; Sandman and Patterson, 2000). Impedance can also fluctuate due to changes in body temperature and because of drying of gel or saline conductors. Measuring impedances throughout the experiment allows data quality to be monitored over longer periods of time and to improve channels with insufficient signal quality during the experiment. However, refreshing the gel/liquid during the experiment may change the signal, possibly introducing additional variance and affecting some analyses. Furthermore, head position tracking systems allow for movement corrections if head restraining methods are not possible, and supine position measures can be useful for future source reconstruction (since MRI is measured in supine position). It should be noted that the positioning of the participant can affect the size of the signals recorded by M/EEG, for example, in supine position, signals from the occipital cortex can increase dramatically (Dimigen et al., 2011) due to reduced amount of cerebrospinal fluid between the brain and the skull (Rice et al., 2013). Co-registered eye-tracking can improve detection and exclusion of ocular artifacts from EEG data.

#### Data analysis

##### Preprocessing

Preprocessing steps such as filtering improve the precision of EEG data by removing high-frequency noise, but can also have unpredictable effects on downstream analyses, affect the temporal resolution of the data, and introduce artifacts (Kulke and Kulke, 2020; Liesefeld, 2018; Rousselet, 2012; Tanner et al., 2015; Vanrullen, 2011; Widmann and Schröger, 2012). We recommend using validated and standardized (semi-)automatic preprocessing pipelines that are appropriate for the nature of the data and the specific research question (see Kulke and Kulke, 2020; Liesefeld, 2018; Rousselet, 2012). If researchers decide to screen for artifacts manually instead, we recommend documenting manual scoring procedures and evaluating inter-rater consistency.

ICA-based artifact removal on strongly high-pass filtered data has been shown to outperform ICA-based artifact removal on unfiltered or less strongly filtered data (Klug and Gramann, 2021; Winkler et al., 2011). Therefore, we recommend creating an appropriately filtered dataset for independent component estimation and transferring the estimated component weights to unfiltered or less strongly filtered data for further processing (Debener et al., 2010; Winkler et al., 2015). Moreover, we suggest using one of several validated algorithms for (semi-)automatic classification of artifactual components (e.g., Chaumon et al., 2015; Mognon et al., 2011; Pion-Tonachini et al., 2019; Winkler et al., 2011). If available, data from external modalities (e.g., measures of heart rate, eye or body movements, video recordings, etc.) can help to identify artifact components showing a high correlation with these variables (e.g., cardioballistic artifacts; Debener et al., 2010).

##### General approach

Most commonly, M/EEG-analyses rely on averaging trials to improve subject level precision, for example, because the size of event-related potentials such as the P300 is small compared to the ongoing EEG activity (see Figure 3B). These averages are then used to extract the dependent variable(s) across the different electrodes that were used and some form of univariate analysis is performed. The flexibility of comparing different electrodes and outcome computations to test the same hypothesis leads to the problem of multiple implicit comparisons (Luck and Gaspelin, 2017). Performing strict Bonferroni correction on all these comparisons would lead to very conservative results that would require unreasonable amounts of data. This can be resolved by correctly identifying familywise error, excluding unnecessary comparisons and performing appropriate multiple comparison correction (see Glossary). Alternatively, mass univariate approaches that explicitly keep the false positive rate at a desired level are well-established (Groppe et al., 2011; Maris and Oostenveld, 2007), but they can make inferential claims less precise (Sassenhagen and Draschkow, 2019). Further, methods have been developed to enable hierarchical modeling of M/EEG-data using GLMs similar to MRI-data, which allows within-subjects variance to be explicitly modeled (Pernet et al., 2011). Even more recently, the power of multivariate approaches for studying brain function using M/EEG has been demonstrated (Fahrenfort et al., 2017; Liu et al., 2021; Schönauer et al., 2017).

##### Source vs. electrode/sensor space analyses

Source space analyses can have higher signal-to-noise ratios than sensor space analyses, often because the process of source localization mostly ignores noise from non-brain areas (Westner et al., 2022). The accuracy of EEG source localization approaches (Asadzadeh et al., 2020; Baillet et al., 2011; Ferree et al., 2001) critically relies on EEG electrode-density/coverage (Song et al., 2015) and the validity of the employed head model, where using the subject’s own MRI scan is recommended over using a template (Asadzadeh et al., 2020; Michel and Brunet, 2019; for more detailed information, see: Gross et al., 2013; Keil et al., 2014; Koutlis et al., 2021; Lai et al., 2018; Mahjoory et al., 2017; Schaworonkow and Nikulin, 2022). Of note, for connectivity analyses performed on EEG data, even if they are performed on source localized data, volume conduction must be considered a source of imprecision that can, however, be overcome (Brunner et al., 2016; Haufe et al., 2013; Miljevic et al., 2022).

##### Time domain analyses

Event-related potentials (Luck, 2005), that is, stimulus-locked averages of EEG activity, are used most frequently in EEG research (Figure 3B). In general, amplitude measures show higher precision than latency measures of ERPs (Cassidy et al., 2012; Morand-Beaulieu et al., 2022). Notably, the measurement error of ERP components varies substantially with the component of interest, the number of experimental trials, and even the method of amplitude/latency estimation (e.g., Cassidy et al., 2012; Jawinski et al., 2016; Morand-Beaulieu et al., 2022; Schubert et al., 2023). Due to this large heterogeneity of precision estimates of ERP measures, routinely reporting subject-level and group-level precision estimates is recommended (Clayson et al., 2021).

##### Spectral analyses

The precision of spectral analyses depends on the method of transferring data from the time to the frequency domain and its fit to the research question (Keil et al., 2014), but more systematic evaluations of the effects of specific methods on precision and data quality are needed. EEG power spectra typically show a rapid decrease of power density with increasing frequencies (He, 2014; Voytek et al., 2015), referred to as ‘1*/f* noise-like activity’. Conventional EEG power spectrum analyses may conflate this activity with narrow-band oscillatory measures (Donoghue et al., 2020). Recent developments offer the possibility to separate aperiodic (1/*f*-like) and periodic (oscillatory) activity components (Donoghue et al., 2020; Engel et al., 2001; Wen and Liu, 2016). Additionally, canonical frequency band analyses may be reported to ensure comparability with previous literature.

### Reporting standards

General guidelines for reporting EEG- and MEG-specific methodological details have been reported elsewhere (Keil et al., 2014; Pernet et al., 2020), but should be followed more consistently by the field (Clayson et al., 2019). One recent suggestion is to calculate the standard error of a single-participant’s data across trials, that is, subject-level precision (Luck et al., 2021; Zhang and Luck, 2023). This summary statistic helps to identify data points (participants or sensors) with low quality. In addition, routinely reporting this statistic may help researchers to identify recording and analysis procedures that provide the highest possible data quality.

### Electrodermal activity (EDA)

Electrodermal activity reflects eccrine sweat gland activity controlled by the sympathetic nervous system (Bach, 2014) which can be recorded non-invasively by electrodes attached to the skin. The signal is composed of a tonic component (i.e., slow variations in skin conductance level; SCL) and a phasic component (i.e., individual skin conductance responses; SCRs). While SCL is related to thermoregulation and general arousal, SCRs reflect stimulus-induced activation (Amin and Faghih, 2021) characterized by different components such as amplitude, latency, rise time, or half recovery time (Dawson et al., 2016). Despite the existence of closely related measures like skin potential, resistance, or impedance, we exclusively focus on skin conductance here, which is measured in microsiemens (µS).

#### Hardware, design, and data recording

Comprehensive overviews and guidelines on data recording are available (Boucsein, 2012a; Boucsein et al., 2012b; Dawson et al., 2016). In brief, the skin should be prepared using lukewarm water (no soap, alcohol, or abrasion) and exact electrode placement should be constant between participants – optimally using anatomical landmarks – to reduce error variance (Christopoulos et al., 2019; Payne et al., 2013; Sanchez-Comas et al., 2021; Boucsein et al., 2012b).

For SCRs, which are rather slow responses, a sampling rate of 20 Hz is considered sufficient but higher sampling rates improve measurement precision (Venables and Christie, 1980). SCRs have an onset lag of approximately 1 s after the eliciting stimulus (0.5 s for high intensity stimuli), which has consequences for the temporal spacing between different experimental events. Responses to temporally close events (i.e., <4 s) are inherently difficult to separate due to the resulting overlapping SCRs with possible consequences for measurement precision. Note, however, that deconvolution-based approaches have been developed for these scenarios (Bach et al., 2013; Benedek and Kaernbach, 2010). Importantly, as novel, surprising, or arousing stimuli elicit SCRs, also events of no interest (e.g., startle probes; Sjouwerman et al., 2016) may result in overlapping SCRs.

Some factors with documented impact on SCRs should also be recorded and controlled including demographic variables like age, sex, or ethnic background (Dawson et al., 2016; Webb et al., 2022) as well as medication or scars at the electrode positions (Christopoulos et al., 2019; Payne et al., 2013; Boucsein et al., 2012b). Furthermore, time of day (Hot et al., 2005) as well as environmental factors like room temperature (Boucsein et al., 2012b) and humidity (Boucsein, 2012a) modulate electrodermal activity and should thus be held constant (e.g., between 20 and 26 °C with a 50% humidity; Christopoulos et al., 2019).

**Figure 4.**
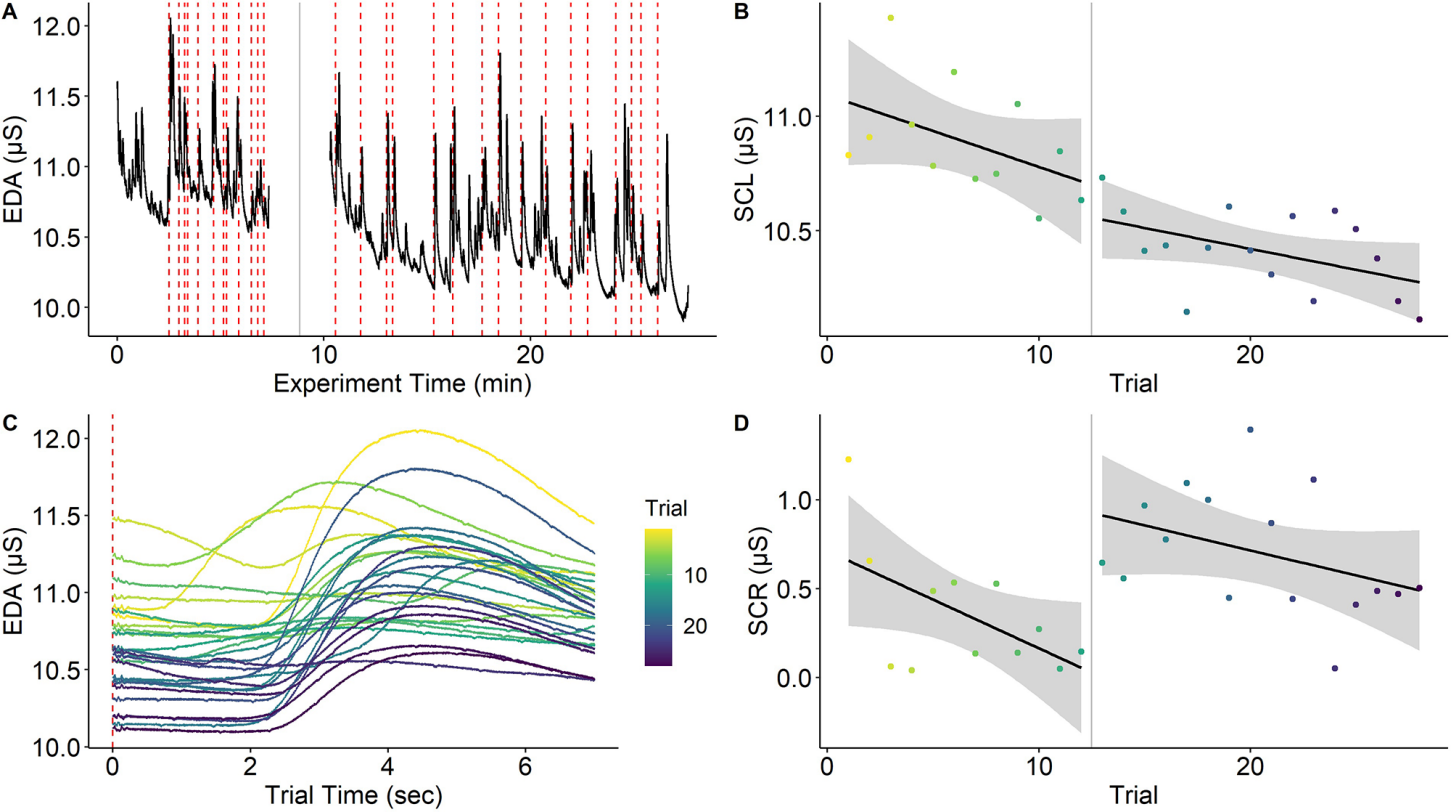
Habituation of electrodermal activity. Habituation of electrodermal activity (EDA) is illustrated using a single subject from Reutter and Gamer, 2023. (**A**) EDA across the whole experiment with the red dashed lines marking onsets of painful stimuli and the gray solid line denoting a short break between experimental phases. (**B**) Skin conductance level (SCL) across trials (separately for experimental phases) showing habituation (i.e., decreasing SCLs) across the experiment. (**C**) Trial-level EDA after each application of a painful stimulus showing that SCL and skin conductance response (SCR) amplitude is reduced as the experiment progresses. (**D**) SCRs (operationalized as baseline-to-peak differences) decrease over time within the same experimental phase. Interestingly, SCR amplitudes ‘recover’ at the beginning of the second experimental phase even though this is not the case for SCL. Notably, this strong habituation of SCL and SCR means that increasing trials for higher precision may not always be possible. However, the extent to which components of primary and error variance are reduced by habituation remains an open question. This figure can be reproduced using the data and R script in ‘Figure 4—source data 1’. Figure 4—source data 1.This zip archive contains EDA data (‘eda.txt’) and rating data (‘ratings.csv’), which are loaded and processed in the R script ‘Habituation R’ to reproduce Figure 4.The R script also contains examples on how to calculate precision for SCL and SCR. The data have been reused with permission from Reutter and Gamer, 2023.

SCRs are subject to strong habituation effects (Lykken et al., 1988; for an illustration see Figure 4). Consequently, increasing the number of trials to augment subject-level precision and reliability (Allen and Yen, 2001) is not straightforward for SCRs. Indeed, larger trial numbers did not generally improve reliability estimates of SCRs (in a learning paradigm; Klingelhöfer-Jens et al., 2022). One interpretation of this result is that increasing precision by aggregation over more trials can get counteracted by sequence effects. Relatedly, habituation must also be considered for within-subject manipulations (i.e., more trials per subject, albeit in different experimental conditions) and weighed carefully against the option of between-subjects manipulations, which may induce interindividual differences in SCL and/or electrodermal responsiveness between groups. Notably, individuals with higher SCL show a higher number and larger amplitudes of SCRs (Boucsein, 2012a; Venables and Christie, 1980). Consequently, adaptive thresholding for SCRs may be a means to increase statistical power (Kleckner et al., 2021).

#### Data analysis

Processing continuously recorded skin conductance data for analysis of stimulus-elicited SCRs requires a number of steps, all with (potential) relevance to measurement precision including response quantification (see Kuhn et al., 2022; Pineles et al., 2009; Sjouwerman et al., 2016), selection of a minimal response threshold (Lonsdorf et al., 2019) with 0.01 µS used as a quite common consensus criterion (Boucsein, 2012a; but see Kleckner et al., 2021 for an adaptive approach), filtering (Privratsky et al., 2020), as well as standardization for between-subjects comparisons (e.g., range-correction; Lykken and Venables, 1971). Few of these steps have been systematically investigated with respect to measurement precision. Recent multiverse-type work suggests that effect sizes and precision derived from different processing and operationalization steps differ substantially despite identical underlying data (Klingelhöfer-Jens et al., 2022; Kuhn et al., 2022; Pineles et al., 2009; Sjouwerman et al., 2016). Furthermore, exclusion of participants due to non-responding in SCRs is based on heterogeneous definitions with potential consequences on measurement reliability and precision (Lonsdorf et al., 2019).

#### Reporting standards

Reporting standards are available (Boucsein et al., 2012b) and include details for subject preparation (e.g., hand washing, skin pre-treatment), data recording (e.g., hard-/software, filter, sampling rate, electrode placement, electrode and gel type, temperature and humidity), data processing (e.g., filter, response quantification details including software and exact settings used, time windows, transformations, cut-offs, non-responder criterion) as well as justifications for the choices.

### Eye-tracking

**Figure 5.**
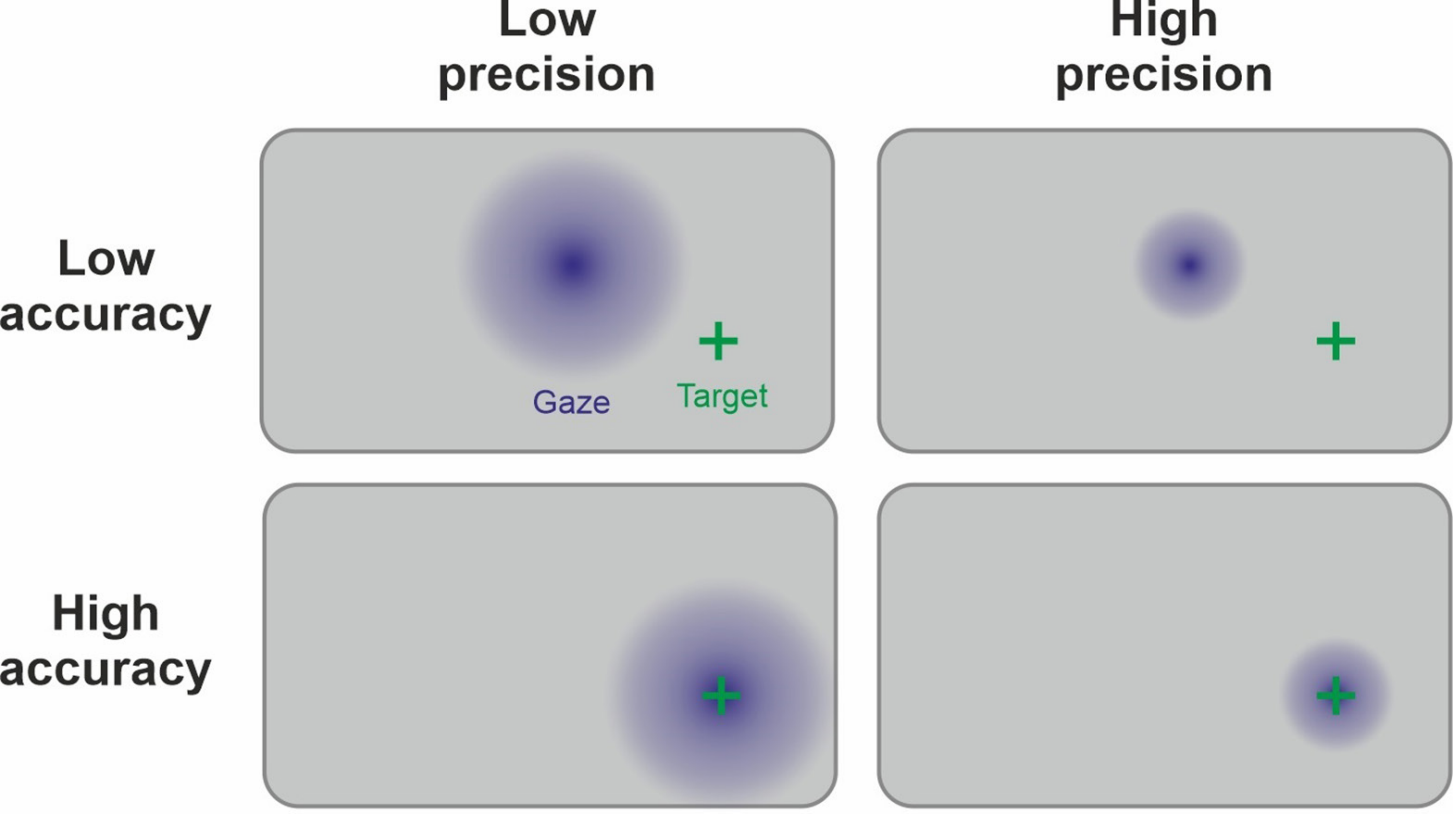
Link between precision and accuracy of gaze signal. Due to the physiology of the eye, the ground truth of the manifest variable (fixation) is known during the calibration procedure. Therefore, accuracy and precision can be disentangled by this step. Accuracy is high if the calibration procedure leads to estimated gaze points (in blue) being centered around the target (green cross). Precision is high if the gaze points are less spread out. Ideally, both high precision and high accuracy are achieved. Note that the precision and accuracy of the measurement can change significantly after the calibration procedure, for example, because of participant movement.

Eye-tracking is the measurement of gaze direction based on the pupil position. We will focus on pupil and corneal reflection methods using infrared light as the currently dominant technology (Duchowski, 2017) but most conclusions are also valid for other applications. Eye-tracking takes an exceptional position in this list of neuroscientific methods as accuracy (Figure 1 or Glossary in Appendix) can be readily quantified as the difference between the recorded gaze position and the actual target’s coordinates (Hornof and Halverson, 2002). Consequently, there is a strong focus on calibration and validation procedures that measure errors of the system (Figure 5). In the eye-tracking literature, ‘precision’ refers specifically to *trial-level* precision (Glossary in Appendix; Holmqvist et al., 2012) of the time series signal during fixations. Another important index of data quality is the percentage of tracking loss, indicating the robustness of eye-tracking across the temporal domain (Holmqvist et al., 2023).

#### Design and data recording

##### Setup-specific factors

Assembling an eye-tracking environment, several factors need to be considered to retain adequate precision. For example, the eye-tracker must have a high sampling rate of at least 200 Hz to prevent an increase in sampling error (Andersson et al., 2010). In addition, distances within a setup should be chosen wisely. Firstly, the operating distance (between participant and eye-tracker) directly affects pupil detection and thus precision and accuracy (Blignaut and Wium, 2014). Secondly, a larger viewing distance (between participant and observed object) decreases the precision of derived measures by diminishing the stimulus image on the retina (i.e., in degrees of visual angle) and thus increases the risk of misclassification in region-of-interest (ROI; also ‘area-of-interest’, AOI) analyses (Vehlen et al., 2022). Since vertical accuracy is usually worse than horizontal, the height-to-width-ratio of the stimuli should also be considered (Feit et al., 2017).

##### Procedure-specific factors

Several factors should be considered prior to data collection. Since accuracy is best in close proximity to the calibration stimuli (Holmqvist et al., 2011), their number and position should be chosen to correspond to the area encompassed by experimental stimuli (Feit et al., 2017). Furthermore, movement of the participant can influence data quality. Although highly dependent on the eye-tracker model, head movement can also affect both accuracy and precision, either through a loss of tracking in remote eye-tracking (Niehorster et al., 2018) or through slippage in mobile eye-tracking (Niehorster et al., 2020). Additionally, a change in viewing distance after calibration can lead to a parallax error (lack of coaxiality of camera or eye-tracker and eyes), threatening the accuracy of the gaze signal (Mardanbegi and Hansen, 2012).

##### Participant-specific factors

Facial physiognomy can affect the quality of the eye-tracking data. For example, downward pointing eye lashes and smaller pupil size decrease accuracy; narrow eyes decrease both accuracy and precision (Blignaut and Wium, 2014) while effects of mascara are debated (Nyström et al., 2013). Data precision of participants with blue eyes was lower than that of participants with brown eye color for infrared eye-trackers (Hessels et al., 2015; Nyström et al., 2013). Visual correction aids influence eye-tracking data quality: Contact lenses decrease accuracy, while glasses decrease precision (Nyström et al., 2013).

### Data analysis

After data acquisition, different analytic procedures have an impact on precision and accuracy. For instance, two classes of event-detection algorithms are available (Salvucci and Goldberg, 2000) to separate periods of relatively stable eye gaze (i.e., fixations) from abrupt changes in gaze position (i.e., saccades): Velocity-based algorithms have a higher precision and accuracy, but require higher sampling rates (>100 Hz). For lower sampling rates, dispersion-based procedures are recommended (Holmqvist et al., 2012). When relying on manufacturers’ software packages, the implemented algorithm and its thresholds are usually not accessible. Thus, systematic comparisons of different procedures are sparse (for an exception see Shic et al., 2008).

After event-detection, additional preprocessing steps can be implemented to ensure high precision for the total duration of the recording. This includes online (e.g., Lancry-Dayan et al., 2021) or offline drift correction procedures (e.g., End and Gamer, 2017) that allow for shifting the calibration map following changes in head position or eye size (e.g., due to tiredness of the participant). Moreover, trials or participants can be excluded during this step based on the proportion of valid eye-tracking data.

Finally, different metrics can be derived from the segmented gaze position data that usually rely on associating gaze shifts or positions to ROIs. A plethora of metrics are used in the literature (Holmqvist et al., 2012) but in general, they describe gaze data in terms of movement (e.g., saccadic direction or amplitude), spatio-temporal distribution (e.g., total dwell time on an ROI), numerosity (e.g., number of initial or recurrent fixations on an ROI), and latency (e.g., latency of first fixation on an ROI). In general, precision is presumably increased for highly aggregated metrics (e.g., dwell time during long periods of exploration) as compared to isolated features (e.g., latency of the first fixation). Some metrics are derived from the raw data prior to event-detection (e.g., microsaccades or smooth pursuit tracking of moving stimuli; Duchowski, 2017). This is mainly due to their infrequent use.

### Reporting standards

Various reporting standards exist and rarely overlap with reporting practices. An empirically informed minimal reporting guideline and an extensive table listing influencing factors on eye-tracking data quality can be found in Holmqvist et al., 2023.

### Endocrinology

Hormones are chemical messengers produced in endocrine glands. They exert their effects by binding to specific receptors (Ehlert and Känel, 2010) and thereby affect various psychological processes (Erickson et al., 2003), which, in turn, may influence hormone concentrations (Sunahara et al., 2022).

Hormones are measured in body fluids and tissues, including blood, saliva, hair, nails, stool, and cerebrospinal fluid. Yet, measures across these measurement domains may reflect different outcomes: While some indicate the current biologically active hormone availability termed acute state (e.g., saliva cortisol), others represent cumulative measures building up over time, termed chronic state (e.g., hair cortisol; Gejl et al., 2019; Kagerbauer et al., 2013; Sugaya et al., 2020; Vining et al., 1983). Critically, samples of different domains often require different sampling devices (Gallagher et al., 2006), handling, and storage conditions (Polyakova et al., 2017; Toone et al., 2013). The adherence to recommendations regarding hormone- and measurement-specific factors is therefore essential to maintain hormone stability, and thus measurement precision (see Resources in Supplementary file 1).

Hormone concentrations are determined with biochemical assays relying on microtiter plates, specific reagents, and instruments. In addition to assay-specific sensitivity and specificity, inter- and intra-assay variation of any given analysis directly relate to measurement precision (El-Farhan et al., 2017). Intra-assay variability refers to the variability of hormone concentrations across identical samples (duplicates) on the same microtiter plate, whereas inter-assay variation refers to the variability across identical samples on different microtiter plates. Many factors can contribute to high variability, such as variation in preprocessing steps (Szeto et al., 2011). Therefore, samples of one study should be analyzed at a single laboratory (ideally in duplicates), with constant protocols, and biochemical reagents from the same manufacturer and charge, thus minimizing variability related to assay components (so-called ‘batch effects’, Leek et al., 2010).

#### Design

A precise measurement of hormone-dependent effects on psychological processes and vice versa requires exact timing of sampling. Often, the collection of hormone samples has to be scheduled with respect to an intervention or event of interest (Stalder et al., 2016) and considering lagged and dynamic hormone responses (Schlotz et al., 2008). Some hormones show early or acute effects on psychophysiological processes that differ entirely from later or delayed effects (Weckesser et al., 2019).

**Figure 6.**
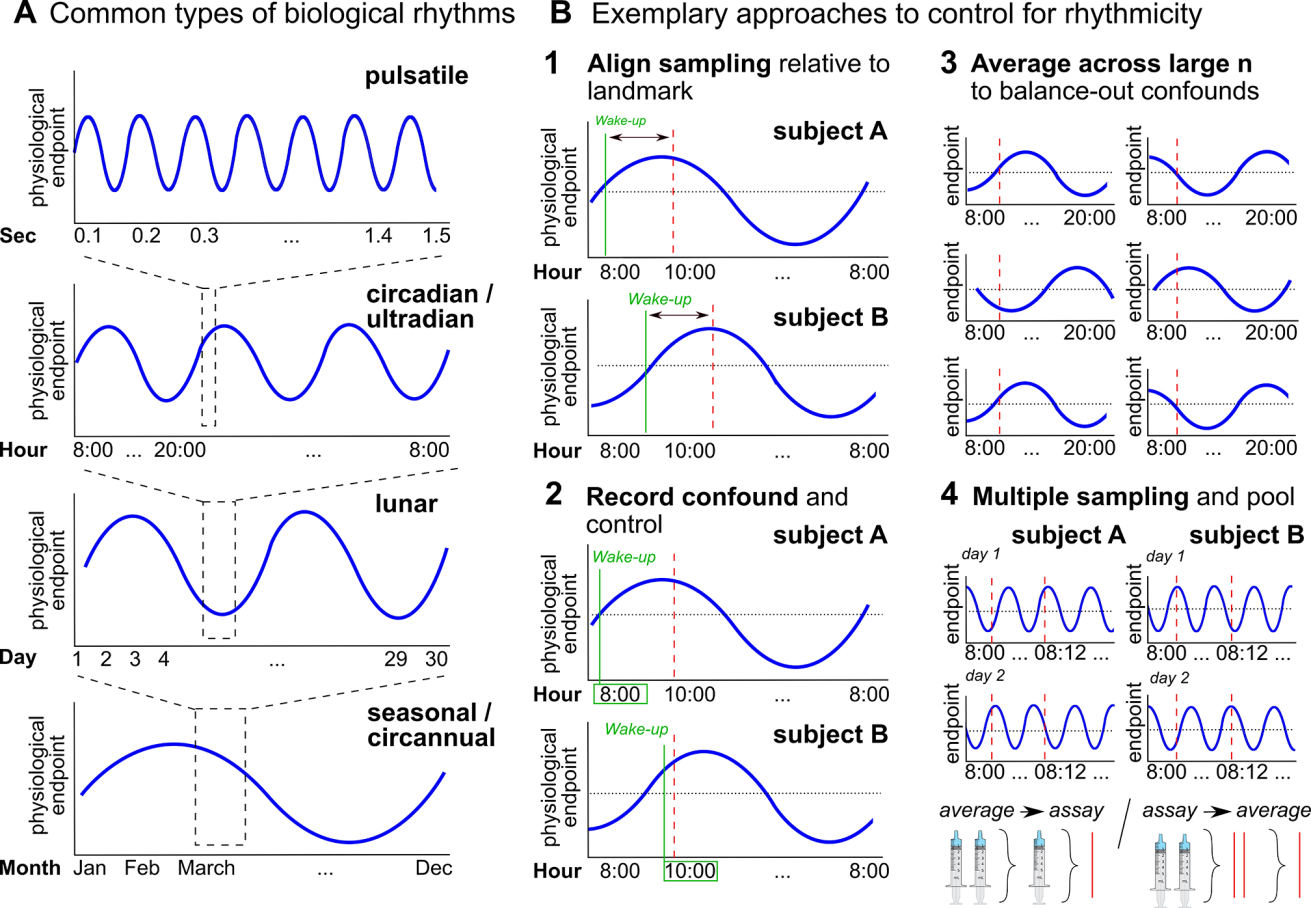
Biological rhythms and how to control for them. (**A**) Examples of biological rhythms. Pulsatile rhythms refer to cyclic changes starting within (milli)seconds, ultradian rhythms occur in less than 20 hr, whereas circadian rhythms encompass changes within a day approximately. These rhythms are intertwined (Young et al., 2004) and included in even longer rhythms, such as occurring within a week (circaseptan), within 20–30 days (lunar; prominent example is the menstrual cycle), within a season (seasonal), or within one year (circannual). (**B**) Exemplary approaches to account for biological rhythms. Time of day at sampling, in itself and relative to awakening, is especially important when implementing physiological measures with a circadian rhythm (Nader et al., 2010; Orban et al., 2020) and needs to be controlled (B1-2). For trait measures, reliability can be increased by collecting multiple samples across participants of the same group, and/or better within participants (B3-4; Schmalenberger et al., 2021).

When hormonal dynamics are considered a confound, collecting hormone samples over multiple time points can increase measurement precision (Sunahara et al., 2022; see Figure 6B, Box 3). However, some hormone concentrations do not necessarily change over a certain time (Born et al., 2002), thereby limiting the utility of additional hormone samples in these cases.

Besides lagged responses, biological rhythms lead to substantial variability in hormone concentrations that may impair measurement precision (Haus, 2007; Figure 6A). While some biological rhythms account for circular hormone changes within just minutes or hours (e.g., circadian rhythm, Figure 6A), hormone concentrations also change within months, seasons, or years (Barth et al., 2015; e.g., puberty or menopause).

Particular attention should also be paid to factors that disrupt biological rhythms. For example, shift work or jet lag typically disrupt diurnal rhythms (Bedrosian et al., 2016), while medication such as oral contraceptives disrupt lunar rhythms and confound physiological endpoints beyond rhythmicity (Brønnick et al., 2020; Fleischman et al., 2010). This additional variation can greatly reduce or even reverse effect sizes (e.g., Shields et al., 2017).

Besides external factors that might disrupt biological rhythms, there are also endogenous shifts in hormone regulation, for example, age-dependent changes related to developmental phases (i.e., puberty and menopause). This variability can confound measures of underlying individual differences in hormonal concentrations. It can be controlled by restricting the target population or by explicitly comparing and statistically accounting for individual development stages. Finally, attention has to be paid to confounds like seasonal fluctuations (Tendler et al., 2021) that impair measurement precision in longitudinal study designs.

Biological rhythms also exist across various modalities including neuroimaging data and receptor activity (Barth et al., 2015; McEwen and Milner, 2017; Orban et al., 2020; Pritschet et al., 2020), with hormones often acting as a driving force (Arélin et al., 2015; McEwen and Milner, 2017; Taylor et al., 2020). Inclusion of hormonal concentrations in statistical analyses can partially control for this variability (e.g., Cheng et al., 2021).

Besides biological rhythms-related confounds, numerous lifestyle and environmental factors affect the variability of hormone concentrations and may limit measurement precision. Although a complete list of potential confounds is beyond the present scope, the most important factors are those with a potential influence on hormone regulation, such as physical and mental health conditions (Adam et al., 2017), medication (Montoya and Bos, 2017), drug, nicotine, and alcohol consumption (Kudielka et al., 2009).

#### Data analysis

Hormone data rarely fulfill the assumptions underlying parametric procedures such as homoscedasticity and normal distribution. Rather than resorting to less powerful non-parametric procedures, data transformations can be used to counteract violations of assumptions (Miller and Plessow, 2013). However, these data transformations must be applied with caution (e.g., Feng et al., 2013). Moreover, hormone data often exist as time series; directly analyzing the repeated measures instead of comparing aggregated scores usually conveys higher analytical sensitivity (Shields, 2020). Time series data further allow the statistical modeling of lagged hormone effects, which can also enhance analytical sensitivity (Weckesser et al., 2019).

Analytical sensitivity can be further increased by building statistical models that capture the nature of hormone effects, which frequently manifest in interaction rather than main effects (Bartz et al., 2011). These effects must be adjusted for potential confounds, either by considering them as factors or covariates in the models. Switching from between- to within-subject designs can also help to increase analytical sensitivity of the models (van IJzendoorn and Bakermans-Kranenburg, 2016), which typically require large sample sizes to be sufficiently powered to detect the effects of interest (Button et al., 2013).

#### Reporting standards

Despite recent calls to improve the rigor and precision in hormone research (e.g., Quintana et al., 2021; Winterton et al., 2021), there is a lack of guidelines describing how hormone findings should be presented (Meier et al., 2022). However, careful documentation of the study design, participant sample with all inclusion and exclusion criteria, type of hormone sample(s) and device(s), time of sample collection, storage procedure with preprocessing steps, and assay type with corresponding inter- and intra-assay variation obtained in the analyses (not the coefficients reported by the manufacturer) is highly recommended.

## Multiple read-out measures

While we have presented precision-related considerations separately for many psychophysiological and neuroscientific methods, it is common to use multiple methods within a single study. Combining different methods allows the assessment of different levels of response, which typically tap into different manifestations of the underlying construct and hence provide complementary insights. For example, it is reasonable to assume that activation in a particular brain area (e.g., the amygdala) precedes and thus predicts a peripheral physiological (e.g., EDA) or behavioral response (e.g., arousal rating). However, there are a number of inherent challenges and specific considerations in combining multiple measures, both in general and in terms of precision.

First, measurement specific idiosyncrasies may impact the to-be-studied process. For example, it has been shown that ‘triggered’ responses, e.g., ratings and startle electromyography (EMG), which require distinct event onsets such as a question or an eliciting tone, can impact the to-be-studied (cognitive) process – for instance by hampering a learning process (Atlas et al., 2022; Sjouwerman et al., 2016).

Second, the recording of multiple measurement modalities may interfere with each other on a purely technical level. For example, when examining SCRs to pictures in combination with tones designed to elicit a startle reflex measured by EMG, the startle evoking tones will not only elicit an EMG blink response but also phasic SCRs. If the sequence and timing of the experimental stimuli (e.g., pictures and tones) are not explicitly tailored to take into account both modalities, they may interfere with each other. The resulting overlap between SCR-responses to stimuli of interest (e.g., a picture) and stimuli of no interest (e.g., tones) may in the worst case preclude meaningful analyses. Similarly, what is a necessary prerequisite for one measurement modality (e.g., eye movements for eye-tracking) may have a detrimental effect on the measurement precision of another measurement modality (e.g., distortion of EEG signals caused by eye movements). Other examples include cardioballistic artifacts in the EEG signal (induced by pulse-related head movements in the magnetic field, Allen et al., 2000, when EEG and fMRI are recorded simultaneously). Similarly, verbal responses during BOLD fMRI acquisition can increase noise in the fMRI signal (Barch et al., 1999). While in some cases it may be possible to correct the signal for such interferences, for example by recording ECG to subtract cardioballistic artifacts from simultaneous EEG/fMRI recordings (Allen et al., 2000), using specific algorithms to detect deviations from the average EEG signal (Allen et al., 2000; Niazy et al., 2005), or independent component analysis (Debener et al., 2007; Mantini et al., 2007), it is usually best to avoid them in the first place through experimental design and specifically tailored experimental timing (e.g., collecting button presses rather than verbal responses in the MRI scanner).

Third, because measurement modalities have inherently different properties, it can be challenging to decide on how to optimize the experimental paradigm to achieve the best possible overall precision. For example, as mentioned above, the gain in precision from increasing the number of trials is not trivial. While increasing the number of participants provides additional independent observations and thus predictable merit, additional trials are subject to sequence effects (e.g., habituation, fatigue, reduced motivation, or learning). For example, while a high number of trials may be beneficial for increasing precision in EEG (Baker et al., 2021; Boudewyn et al., 2018; Chaumon et al., 2021), such a high number of trials may decrease precision in EDA due to strong habituation of SCRs. For example, to capture responses prone to habituation, ‘dishabituation’ can be achieved by adding novel stimuli (Sperl et al., 2021). Another solution could be to pre-register the optimal number of trials per measurement modality and only include this number of trials in subsequent analyses. However, this may not be feasible for studies with a learning element as early and late trials may tap into different stages of a process that is expected to change over time (Sperl et al., 2021).

Fourth, because different measures inherently differ in precision they also differ in statistical power. Using behavioral performance as the basis for calculating power and sample size estimations for neuroscientific methods is likely to be misleading. This might result in underpowered studies that threaten scientific progress (Button et al., 2013).

Fifth, when investigating associations between two different measures, it is important to keep in mind that the precision of the least precise measurement determines the upper boundary of an observable relationship. More specifically, the correlation between two variables cannot exceed the smallest reliability exhibited by any of the two variables (Spearman, 1910). Using multiple read-out measures in a single study comes with the inherent challenge of determining whether the same or different hypotheses exist for different measurement modalities and, in the former case, the extent to which that hypothesis can be considered confirmed if only one of these modalities shows the expected effect. In fact, such divergent findings may be related to precision being optimized for one measurement modality but less so for another. Furthermore, in the case of different predictions for different measurements, a correction for multiple comparisons is generally not necessary (Feise, 2002), whereas controlling for Type I (alpha) error may be warranted if the hypothesis is considered to be confirmed if the effect is observed in one out of several outcome measures.

Sixth, pseudo-relationships between two measurements can arise from related secondary variance between these measurements. For example, head movement may simultaneously affect both EEG and MRI measures, leading to similarities in their signals. These could introduce spurious correlations between the EEG and MRI data even in the absence of a meaningful conceptual relationship between them (Fellner et al., 2016).

Seventh, when analyzing time series data from multiple neuroscientific measurement modalities together, it is important to allow for precise synchronization in the time domain during acquisition. This is easier to achieve if the two signals are acquired by the same device (e.g., EEG and EOG by the same amplifier, or MRI and peripheral physiology by the same MRI scanner). If this is not possible, precision can be optimized by ensuring that all devices are synchronized in clock time (Bullock et al., 2021; Mandelkow et al., 2006; Xue et al., 2017). Software solutions for device synchronization exist (e.g., Lab Streaming Layer) and are being augmented by efforts to provide low-cost hardware solutions (Bilucaglia et al., 2020). This issue is also very important to consider when performing hyper-scanning studies (Babiloni and Astolfi, 2014; Barraza et al., 2019). In addition, precision can be further improved by considering brain time instead of clock time to synchronize neuroscientific measurements between participants according to ongoing oscillatory brain dynamics (van Bree et al., 2022).

## Discussion

As we have argued throughout this review, the precision of psychophysiological and neuroscientific methods is affected by a number of technical, procedural, and data analysis steps. Increasing precision improves the estimation of statistical effects, largely independent of the costs associated with increasing sample size. This has important implications for how we conduct and evaluate power analyses, as several aspects beyond sample size must be considered and compared across studies when basing a power analysis on previous research: How were measurements protected from unsystematic influence? Was similarly precise technical equipment used? Were appropriate designs and robust participant preparation procedures applied? Is the number of trials comparable across studies? What preprocessing steps were taken to decrease noise? What covariates were recorded and included in the analysis? Critically, the exact extent to which these steps impact precision and statistical power is currently largely unknown and needs to be systematically evaluated in the future.

### Planning for precision at the level of interest

One advantage of considering measurement precision is the explicit reference to levels of aggregation: Are we studying *group-level differences* or *associations with subject-level estimates*? Within this framework, it becomes more intuitive how different research questions require precision at their respective levels of aggregation (i.e., group- or subject-level).

More specifically, different optimizations of certain variance components may come in handy: For group differences, reducing between-subjects variance (within the same groups) increases precision at the group level for a given sample size (Hedge et al., 2018). For correlational hypotheses, however, the between-participant variance should be maximized to stabilize the relative positions between subjects (given constant subject-level precision, Figure 2). Consequently, the ‘two disciplines of scientific psychology’ (Cronbach, 1957), that is, experimental psychology and correlational psychology in Cronbach’s terms, require different optimization strategies with respect to between-subjects variance.

**Figure 7.**
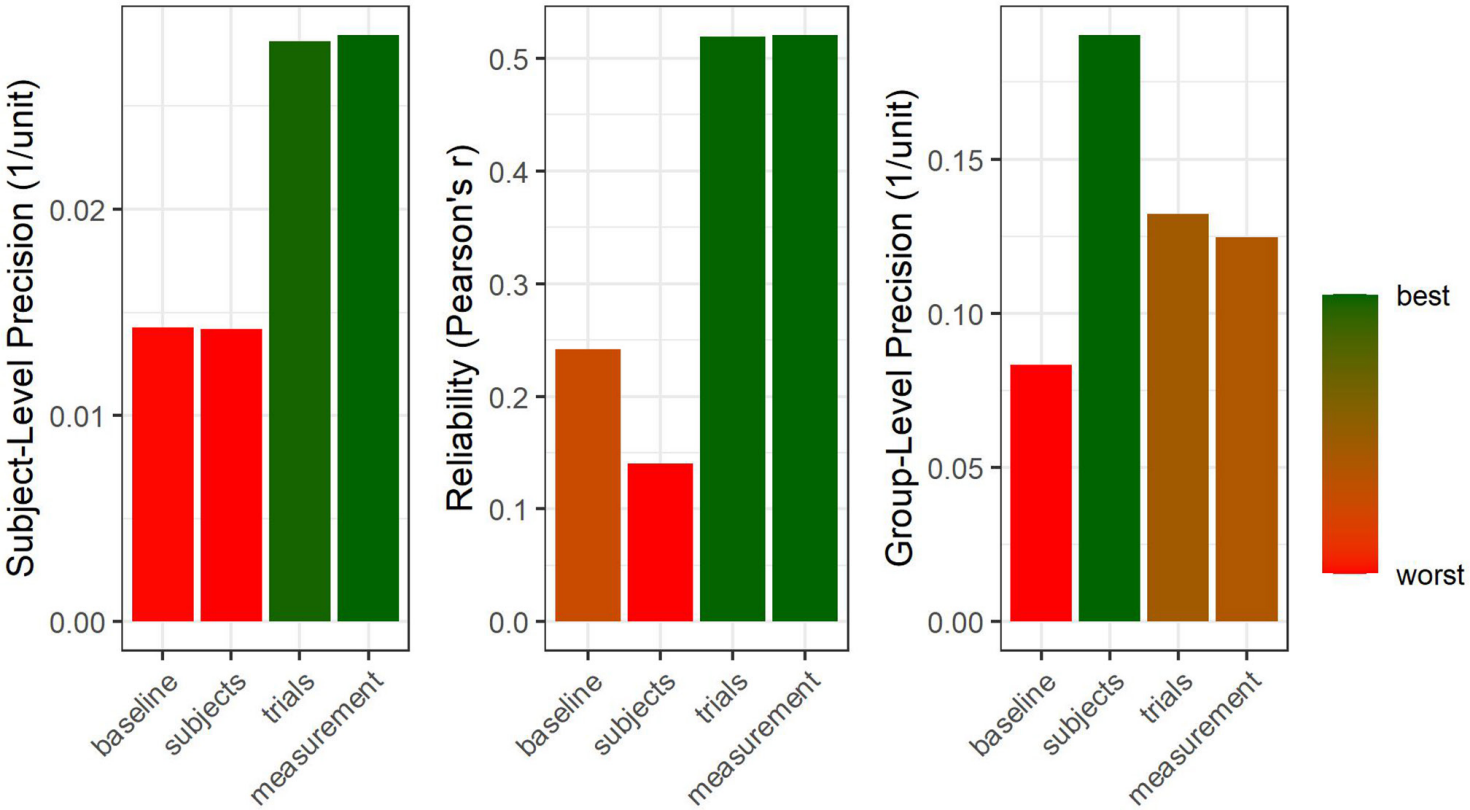
Hierarchical structure of precision. Four samples were simulated at different degrees of precision on group-, subject-, and trial-level. We start with a baseline case for which all levels of precision are comparably low (64 subjects, 50 trials per subject, 500 arbitrary units of random noise on trial-level). Afterwards, the number of subjects is quadrupled to double group-level precision (right panel) but no effect on subject-level precision or reliability is observed (a descriptive drop in reliability is due to sampling error). Subsequently, the number of trials is quadrupled to double subject-level precision. This also increases reliability and, vitally, carries on to improve group-level precision (Baker et al., 2021), albeit to a smaller extent than increasing sample size by the same factor. Finally, the trial-level deviation from the true subject-level means is halved to double trial-level precision. This improves both subject-level and group-level precision without increasing the number of data points (i.e., subjects or trials). Figure 7—source code 1.This R code can be used to reproduce Figure 7.

Less controversial, on the other hand, is the role of within-subject variance: This component should usually be minimized to increase the precision at the subject-level. For correlational hypotheses, this decreases error variance by definition (Glossary in Appendix: Reliability). For group differences, high subject-level precision carries on to improve group-level precision (Baker et al., 2021), providing a win-win scenario for statistical power and reliability. Indeed, precision can be viewed as a one-way street, with trial-level precision carrying on to improve subject-level precision, which in turn improves precision at the group aggregation level (see Figure 7). To this end, the merit of increasing sample size is strictly limited to group-level precision, with no benefit to subject-level estimates or reliability. Consequently, in the absence of further information on the efficiency of increasing precision on different levels, priority should be given to optimizing trial-level precision.

### Systematically evaluating measurement precision

So far, we have shown that different decisions during study design and data analysis affect measurement precision. However, there is currently very little information on the size of this influence and thus on how efficiently precision can be improved by adopting different strategies. A first step in addressing this lack of information is to routinely report measures of precision in quantitative studies. While it is becoming standard practice to include indices of group-level precision by reporting confidence/credible intervals around effect size estimates, this is rarely the case for measures of subject-level precision or reliability. In our view, the main obstacle to quantifying subject-level precision is the need for trial-level data (Parsons, 2021), whereas most researchers are formally trained to deal statistically with subject-level aggregates only while using external programs or scripts for preprocessing of trial-level data. Thus, developers of preprocessing toolboxes are called upon to include metrics of precision in their software (e.g., as currently implemented in the ERPLAB toolbox, Lopez-Calderon and Luck, 2014). In the simplest case, precision can be quantified by calculating the standard error at each aggregation step (“Figure 7—source code 1”). For more complex preprocessing strategies such as first-level analyses or computational modeling, methods need to be refined or developed.

A promising way to systematically quantify the impact of different choices during data analysis on precision is to employ multiverse (Del Giudice and Gangestad, 2021; Steegen et al., 2016) or specification curve (Simonsohn et al., 2020) analyses. For research questions targeting group differences, group-level precision should be investigated, whereas for correlational hypotheses, subject-level precision is the outcome of choice (Zhang et al., 2023; Zhang and Luck, 2023; for an example on reliability, see Parsons, 2020; Xu et al., 2023). As little is known about the potential differential precision of different outcome measures, it is important to address this issue across various measures and paradigms (Klingelhöfer-Jens et al., 2022). For example, the complex relationship between trial number and precision should be further explored across different outcome measures, as sequence effects complicate the influence of increasing trial number on precision estimates due to habituation effects and fatigue counteracting the benefit of additional observations (see EDA vs. EEG, for example). This can be investigated empirically by including trial number as a specification parameter in a multiverse or specification curve analysis. The results can be used to guide design decisions regarding the trade-off between spending additional resources to increase sample size or number of trials. Importantly, multiverse approaches require careful selection of the options included (for a critical discussion see Del Giudice and Gangestad, 2021). To this aim, we encourage researchers to routinely quantify and report both reliability and precision estimates (e.g., via confidence/credible intervals around effect sizes). When creating figures, we recommend visualizing the variance between subject-level estimates for group-level differences beyond simple bar or line plots (e.g., using rain cloud plots; Allen et al., 2019; Weissgerber et al., 2015). Subject-level precision can also be illustrated using error bars around individual data points, which is particularly useful for scatter plots representing correlational hypotheses (Source Code Files ‘Reliability, between and within variance’ and ‘Figure 7—source code 1’).

### WEIRD challenges

Human neuroscience aims to study the human mind and its biological correlates in general. However, as noted above, precision at the group-level can be increased by minimizing the standard deviation of subjects within a group. For this and other reasons (such as convenience), research on homogeneous sub-populations of young, right-handed, neurotypical, white individuals (cf. the acronym WEIRD: ‘Western, Educated, Industrialized, Rich, Democratic’; Henrich et al., 2010) has often been favored in human neuroscience and beyond. While women are often excluded for certain research questions, for example due to the effects of sex hormones (Criado-Perez, 2019), convenience sampling in psychology often results in predominantly female samples (Weigold and Weigold, 2022). This issue is further complicated by the need to distinguish between sex and gender (Hines, 2020). To improve precision, the participants should always be asked subjectively about their sex assigned at birth and their current gender identification (National Academies of Sciences, Engineering, and Medicine, 2022). Researchers might also consider using dimensional scales (e.g., ask the participants to rate themselves on two independent scales of masculinity and femininity) or collecting objective measures such as sex hormone levels rather than relying on categorical self-classifications of sex and gender. Selective exclusion of any subgroup – for example in the name of precision – must be carefully weighed against the loss of generalizability of the results. It should be noted that collecting a more representative sample (as opposed to a homogeneous one) always enhances the generalizability of results. However, it does not necessarily mean that modulations of the effect by different groups can be detected if the study does not have sufficient statistical power (Brysbaert, 2019; Maxwell et al., 2017; Sommet et al., 2022). In addition, the reliability of the measures may be attenuated if there is insufficient between-subjects variance, limiting the use of this measure for subject-level investigations (Hedge et al., 2018).

These challenges can also be viewed through the lens of the artificial nature of the laboratory situation and the limitations of data collection to privileged communities living close to advanced research facilities. Ambulatory assessment has become popular to overcome these issues. While MEG has begun to overcome the need for super-cooled sensors, it remains limited to a magnetically shielded room (Tierney et al., 2019). Structural MRI scans can potentially be assessed using low field MRI machines that can be built into a cargo van (Deoni et al., 2022) and mobile imaging of the cortical BOLD signal is possible using functional Near Infrared Spectroscopy (fNIRS), even in areas as remote as rural Africa (Lloyd-Fox et al., 2014). Beyond these somewhat extreme examples, researchers’ ability to perform eye-tracking and EEG in settings closer to everyday life has advanced significantly (Debener et al., 2015; Goverdovsky et al., 2017; Rösler et al., 2021). In general, such methodological advances are to be welcomed as they allow the study of a wider range of situations and individuals, as well as potentially increasing sample sizes. However, it is equally important to ensure that these methods provide sufficient precision, to avoid offsetting these benefits, as they come with their own drawbacks (e.g., motion artifacts in mobile EEG).

Crucially, the neglect of reliability and precision at the subject-level has handicapped human neuroscience in terms of its translation into (clinical) application (Ehring et al., 2022; Lachin, 2004; Moriarity and Alloy, 2021). We anticipate that addressing this gap will enhance the applicability of human neuroscience by establishing results that are meaningful at the individual level, rather than only at the group level. Importantly, excluding participants with the aim of reducing variance by homogenizing the sample should not be done on the basis of rules of thumb but must be supported by empirical data, showing that the excluded variability is not primary variance (see Figure 3A). Moreover, we argue that such variance is better dealt with by statistical approaches (e.g., treating it as secondary variance through covariates) and encourage researchers to embrace diversity (i.e., high inter-subject variability). Beyond these precision-related considerations, human neuroscience also has a moral obligation to study representative samples and to produce findings that are generalizable to all humans.

### Improving measurement precision in future research

Information on the precision of measurements is not usually included in project proposals and outlines. In part, this may be due to a lack of available knowledge about these specifics or their impact on statistical power and the interpretation of results. In fact, even sample size or power calculations are still rarely reported in some areas of neuroscience. In neuroimaging research, sample sizes remain small and appear to have increased only slowly over the past ten years (Szucs and Ioannidis, 2020). While statistical power in neuroimaging has received more attention over the past decade (Button et al., 2013; Marek et al., 2022), only 3–4% of studies published in 2017/2018 included a-priori power calculations and 65% did not mention power at all (Szucs and Ioannidis, 2020). Ensuring precise measurement at the individual level is central to reducing error variance and thus increasing the likelihood of identifying a true positive effect (see Figures 2 and 3). Precision can be directly improved through careful study design and often involves little additional costs, unlike other determinants of statistical power.

This also highlights the importance of careful piloting and task evaluation, as opposed to the use of unvalidated tasks. Indeed, the development of new task paradigms is an integral part of the research process that requires careful validation steps that estimate and report precision, reliability, validity, and effect sizes. However, it is often difficult to obtain funding for task validation studies, so there are often few resources available for this central part of the research process. Additional funding schemes may therefore be needed to support task development and validation. In general, information on precision and reliability should be a fundamental part of reporting and should be shared together with the code to run the task for future applications to provide a more thorough basis for subsequent power analyses and study design. This should therefore be strongly encouraged by reviewers of grant applications and journal articles, publishers, and funding agencies. In this context, we also highlight the value of exploratory analyses and urge reviewers and funders to take this into account more in the future (Scheel et al., 2021).

Importantly, effect sizes derived from the literature are often inflated due to publication bias, which favors studies with small-samples that happen to show strong effects (Button et al., 2013; Schäfer and Schwarz, 2019; Szucs and Ioannidis, 2017). Therefore, when planning a study or applying for funding, a conservative approximation of the true effect should be considered, for example, by using the lower bound of the 95% confidence interval of a published effect. Public sharing of research data and adherence to common reporting standards will have an indirect impact on measurement precision, as the availability of a larger body of empirical and reusable data will allow for a more aggregated and less biased estimation of effect sizes, the exploration of their determinants, and the assessment of the impact of procedural and statistical choices, with the goal to guide informed decisions for future work. This will facilitate statistical power analyses, which are essential for conducting conclusive yet cost-efficient studies. In particular, data sharing also facilitates collaborative efforts such as data pooling and mega-analyses, which can also focus on effects of interest that are too small to be studied with sufficient statistical power by a single research team. If the required sample size is still too large despite optimization of measurement precision, large-scale consortia (e.g., Thompson et al., 2014) are essentially required.

In summary, we highlight the importance and prospects of publicly sharing primary and secondary data and analysis code whenever possible. We argue that this must become a natural part of the (neuro-)scientific research process as it supports cumulative science (also in terms of measurement precision). However, we also emphasize the key role of ensuring the (re-)usability of publicly available data through the provision of adequate meta-data and the use of standardized formats. The Brain Imaging Data Structure (BIDS) standard (Gorgolewski et al., 2016; https://bids.neuroimaging.io/specification.html) has already been adapted for this purpose for a variety of outcome measures such as EEG (Pernet et al., 2019) and MEG (Niso et al., 2018) and its use is highly recommended (see Table of Resources in Supplementary file 1).

As measurement precision is one of the key determinants of statistical power, we hope that our review will provide useful resources and synergies that help to increase future consideration of both measurement precision and its implications for statistical power.

### Conclusions and future directions

In general, methods to improve precision are a valuable addition to the researcher’s toolbox. However, to take advantage of these methods, researchers need to have sound information about the factors that contribute to precision. In this primer, we provide an up-to-date overview of the topic and direct the reader towards valuable resources. However, many open questions remain. To relate different measurement methods to each other with confidence, it is crucial to be able to evaluate their respective precision empirically, rather than basing neuroscientific research on implicit and often vague assumptions about sufficient precision. Therefore, researchers should report empirical estimates of the precision achieved (see above). In addition to standardized effect sizes, it is essential to report the different variance components, for example in the form of precision estimates. In addition, calibration experiments (Bach et al., 2020), which aim at facilitating the optimization of measurement strategies and the quantification of measurement uncertainty, constitute a promising approach. Such standardized calibration experiments or field-specific datasets could also be used to build up a large database and systematically assess different contributors to measurement precision through large-scale mega- and/or meta-analyses (Ehlers and Lonsdorf, 2022) as well as multiverse approaches (Parsons, 2020). Such an approach may seem tedious in the short term, but we are convinced that it will lead to a more robust and resource-efficient human neuroscience.

## Additional files

Supplementary file 1. Resources.

## Appendix 1

In the glossary, we define core concepts relevant to this review. We acknowledge that these definitions may vary between different disciplines in human neuroscience.

### Types of Variables

#### Dependent and independent variables

For group differences, *dependent variables* are outcome measures that are affected by a (quasi-)experimental manipulation. *Independent variables* are the variables that can be manipulated or controlled by the experimenter.

For correlational hypotheses the independent variable is called the *predictor* and the dependent variable is the *criterion*. In scenarios with observed predictors (i.e., no direct manipulation of predictors), no causal inference can be drawn, rendering predictors and criterions interchangeable.

#### Covariates

*Covariates* are variables that affect the dependent variable but are possibly not of central interest for the researcher (e.g., age, sex, or gender). They are included in the statistical model to reduce unexplained variation and thus decrease error variance (see “Variance and its components”).

#### Latent and manifest variables

Most human neuroscience research is interested in *latent variables*, i.e., theoretical psychological processes or constructs that cannot be measured directly (e.g., emotion processing). *Manifest variables* that can be measured directly (e.g., the magnitude of startle responses) are then used as *operationalizations* of these latent constructs. The relationship between the latent and the manifest variables (i.e., the *construct validity*) is often unknown but may be estimated with psychometric methods such as latent variable models (Borsboom et al., 2003).

#### True score

According to classical test theory, an observed (i.e., manifest) test score can be decomposed into a *true score* and an error component. The true score is the theoretically expected value of the manifest variable over an infinite number of independent observations. Hence, we use the concept of true scores in this manuscript to discuss the *accuracy* (see below) of particular operationalizations.

### General linear model (GLM)

A statistical model seeking the linear relationship between the independent variable(s) and the dependent variable(s) that minimizes the residual error and can be written as the linear equation *Y*_*n*k*_ *= X*_*n*k*_*B*_*m*_ *+ E*. *T*-tests, analyses of variance (ANOVAs), correlations, and linear regressions can be understood as special cases of the general linear model including fixed effects only (see below). For example, the *t*-test can be written as *Y*_*n*_ *= X*_*n*_*B*_*1*_ *+ B*_*0*_ *+ E*, where *Y* is the dependent variable, *X* is the dichotomous independent variable, *B*_*1*_ is the slope (i.e., the effect) between the two factor levels, *B*_*0*_ is the intercept, and *E* is the residual error.

#### The GLM in neuroimaging research

In human neuroscience, there are often several independent variables and covariates that predict many dependent variables (e.g., activation pattern of several thousand voxels in the brain). Thus, neuroscience showcases the utility of the GLM. In general, matrix *Y* represents the dependent variable(s). *X* is often called a *design matrix* because its columns encode the variables of the study design (e.g., the different stimuli). Matrix *B* contains the *regression parameters* and is estimated to minimize the error matrix *E*, i.e., the deviations of the predicted from the actual values of the dependent variable(s), also called *residuals*.

#### Variance and its components

The most commonly calculated index of variance is the mean squared difference of the measurements from their sample mean. The square root of the variance is called the *standard deviation* (SD).

In the GLM, the total variance of a dependent variable is partitioned into different components: *Primary variance* is predicted by changes in the independent variables, *secondary variance* is accounted for by changes in covariates, and *error variance* is the remaining unaccounted variance of the residuals (see Figure 2).

#### Difference scores

Some methods of human neuroscience rely on differences between conditions (e.g., contrasts in fMRI or modulations of ERP components in EEG). In this case, the variance of the difference scores across subjects is equal to the sum of the variances of contributing scores minus their covariance. This means that difference scores reduce between-subjects variance if the two observations are highly (positively) correlated and increase variance if they are not (or highly negatively) correlated. Therefore, if the scores are positively correlated, the reliability of their difference is often lower than the reliability of either single observation (due to reduction of between-subjects variance, see Figure 2).

#### Variance between and within subjects

*Within subject variance* is estimated by the variability across trials of the same subject within the same condition(s) in one experiment. This variability should usually be minimized to increase subject-level precision. Within subject variance is often disregarded in statistical analyses due to *aggregation* (see below), i.e., the creation of *subject-level estimates*, which are then submitted to a GLM. However, this type of variability carries on to the subsequent level of aggregation and thus increases estimates of between subjects variance (see next paragraph). Consequently, accounting for within subject variance using a General Linear Mixed Model (see below) usually increases group-level precision by decreasing the estimate of between subjects variance.

*Between subjects variance* is defined as the variability of the measurement between the true scores of participant averages (of the same group) and thereby describes the heterogeneity of the sample(s). For *group differences*, small variance between subjects (of the same group) is beneficial for statistical power by increasing *group-level precision*. For *correlational hypotheses,* however, systematic variance between subjects should be maximized (Discussion). Between subjects variability is often operationalized as the variance of the *observed* subject-level *estimates*. Critically, this leads to an overestimation since variability between trials of the same subject also contributes to the observed variance of subject-level estimates (Baker et al., 2021; Penny and Holmes, 2007). Thus, paradoxically, the *observed* variance between subject-level estimates does not purely reflect the heterogeneity of the sample but includes within subject variance to a certain degree. General Linear Mixed Models (see below) can account for this bias by decomposing the variance of the upper level into its contributing factors: true score variability between subjects and within subject variance carrying over to inflate the variability of subject-level estimates.

#### Systematic and random error

*Systematic error* describes error variance that is correlated with an independent variable and hence confounds the attribution of effects of the independent variable on the dependent variable (e.g., differences in luminance between stimuli with positive and negative emotional content). In contrast, *random error* has no specific relationship to independent variables (included in the statistical model). This makes the statistical conclusion less precise but does not bias it in a particular direction (e.g., poor electromagnetic shielding). In the GLM, the error variance of matrix *E* is always assumed to be random.

#### Aggregation

Aggregation is the process of increasing precision by integrating repeated measures into a single value of interest. For *point estimates*, usually the arithmetic mean is used. The error variance around the arithmetic mean is called *standard error* (SE) and is estimated by the standard deviation (SD) of the observations divided by the square root of the number of observations: SE = SD / √n.

#### Effect size

An effect size is used to describe an observed effect by a single number. This can be done in units of the measurement (i.e., *unstandardized effect sizes*) but cannot be easily interpreted without knowledge of the underlying variance. *Standardized effect sizes* can be calculated for group differences (e.g., Cohen’s *d* or partial eta squared η_*p*_^2^) and for correlations (e.g., Pearson’s *r*) by normalizing the unstandardized effect size by the variance associated with this effect. For Cohen’s *d*, the normalization procedure involves dividing the absolute difference by the standard deviation of the subject-level aggregates, so that small standardized effect sizes can be due to small absolute differences or large variance. This means that very small effect sizes may indicate a lack of precision or a small effect (or insufficient mapping of the latent on the manifest variable). For Pearson’s *r*, the normalization procedure involves dividing the covariance by the multiplied standard deviations. Hence, small effect sizes can be due to low covariance or large standard deviations. In ANOVAs in which covariates or additional predictors are included, the primary variance is normalized against the remaining error variance after accounting for secondary variance. Here, a small effect size can be due to a small amount of explained variance by the predictor (i.e., primary variance) or due to a model that has not accounted for all sources of secondary variation, which increases error variance (see Figure 3).

#### Fixed effects

We define fixed effects as effects that are the same for the entire sample or group (see definition 1 in Gelman, 2005 but also mind other definitions). This means that a certain manipulation is assumed to change the ERP by exactly 15 µV for all subjects. All deviations from this effect are either secondary variance or error variance, but no differences between subjects in their reaction to the manipulation are expected.

#### Random effects

Random effects extend fixed effects inasmuch as they allow estimating deviations of an effect within a participant from the sample’s estimated fixed effect. For example, a certain manipulation may change the ERP by 10 µV in one participant and by 20 µV in another participant. This is implemented in the general linear mixed model (see below) by assigning different regression parameters in the matrix *B* to different subjects (i.e., *random slopes* or *random intercepts*). To include random effects, repeated measures need to be applied (i.e., several trials of the same participant in the same condition). Without random effects, the individual’s deviation from the group-level fixed effect cannot be modeled and adds to error variance.

### General linear mixed model (GLMM)

General linear mixed models (also “linear mixed effects models”) are extensions of GLMs. They entail the associations between independent variables (predictors) and a dependent variable (criterion) as so-called fixed effects in classical regression models. In addition, GLMMs comprise so-called random effects, that is, variations across subjects, stimuli, or other grouping variables (see above). Therefore, mixed effects models allow analyzing data on a trial-by-trial level due to their hierarchical setup and can thus decrease error variance by identifying additional components of secondary variance (e.g., non-random fluctuations within subjects). As mixed effects analyses are applied prior to aggregation of observations across trials of the same subject, they are based on more information and, thus, usually achieve greater statistical power given the same raw data.

### Related concepts in human neuroscience

#### Preprocessing

Preprocessing describes all procedures that are performed to transform the raw data into data that can be analyzed using inferential statistics (e.g., a GLM). The precise preprocessing steps vary between research methods (e.g., filtering in M/EEG research or alignment in MRI research). Most preprocessing pipelines contain a step of artifact rejection, which uses automatic, semi-automatic, or manual procedures to discard parts of the data that are assumed to contain an insufficient ratio of signal to noise or physiologically implausible values. The multitude of preprocessing steps and (in many cases) the absence of a clear gold standard implies that a variety of different preprocessing pipelines exist that can influence the outcome of the inferential statistics and the interpretation of the study.

#### First and second level analysis

To improve the computational efficiency of analyzing fMRI data, a common approach in fMRI research first fits a GLM for each individual subject to model the individual BOLD time series as a function of the experimental conditions (first level analysis). Then the estimated parameters are used as new data to fit a GLM at the group level whose estimated beta coefficients often reflect the mean across individual data (second-level analyis; Penny and Holmes, 2004; Poline and Brett, 2012). This step-wise approach leads to similar results as an “all-in-one” GLMM (see above) if proper corrections for correlated errors and unequal error variances are performed (non-sphericity correction; for details see Beckmann et al., 2003; Friston et al., 2005).

#### Mass univariate analyses

Many methods used in human neuroscience yield many measurements per participant (e.g., voxels, electrodes, frequency bands, genes) that are often analyzed using one univariate test per measurement. This is in contrast to using multivariate analyses (or additional factors) to analyze these measures together.

#### Multiple testing correction

Without accounting for multiple testing, mass univariate approaches would produce many falsely positive results. Overly conservative correction of multiple dependent comparisons (e.g., Bonferroni correction), however, would yield many false negatives. A middle ground is provided by algorithms such as False Discovery Rate (Benjamini and Yekutieli, 2001) or (cluster-based) permutation tests (Maris and Oostenveld, 2007). Cluster-based tests take the spatial structure of data into account when correcting for multiple comparisons, e.g., voxels or electrodes that are close together are expected to show correlated activation (Worsley et al., 1992).

#### Data simulations

Simulations are formal models of the data generating process that can be used to determine the power for complex study designs. Often, they invert a statistical model to generate data by choosing coefficients on the basis of previous findings. For example, starting from a GLM of the form *Y*_*n*_ *=* 0.5*X*_*n*_ *+* 1 *+ E* (i.e., setting *B*_*1*_ = 0.5 and *B*_*0*_ = 1), one would generate values of *X* per participant (e.g., two conditions 0 and 1), multiply them by the coefficient 0.5, add the intercept 1, and then add a value *E* randomly selected per participant.

### Precision and related concepts

#### Precision

*Precision* of the measurement in this review is defined as the ability to measure the manifest variable repeatedly with as little variability as possible (given a constant true score) at an aggregation level of interest (Cumming, 2014; Trajković, 2008). Thus, it is readily operationalized as the inverse of the corresponding standard error: Precision = 1/SE = √n / SD. Consequently, high precision can be achieved by increasing the number of independent observations or by minimizing the estimated standard deviation across them. The latter, in turn, can be obtained by decreasing uncontrolled influences on the measurement (e.g., via shielding) or by accounting for systematic influences via covariates. Specifically, the two most important types are *group-level precision* (1/SE across subject-level aggregates), which is affected by the number of subjects and the homogeneity between them, and *subject-level precision* (1/SE across trials, i.e., one for each subject; Luck et al., 2021) that is determined by the number of trials and variability between trials (within the same subjects). Importantly for the latter, changes in true scores across repetitions (e.g., due to *sequence effects* like habituation, fatigue, or learning) need to be modeled or they will decrease subject-level precision in a way that cannot be ameliorated by additional observations of the same subject. For time series data, *trial-level precision* can be computed (1/SE across repeated measures within a single trial; Eye-Tracking section of the main article).

Related concepts to precision are *signal-to-noise ratio*, *reliability*, *accuracy*, and *validity*. All these constructs can be improved by decreasing error variance, but each one defines noise differently.

#### Signal-to-noise ratio

*Signal-to-noise ratio* is a generic term denoting any separation of variance into a primary (“signal”) and an error (“noise”) component and calculating their quotient. Test statistics like *t*- or *F*-values can be interpreted as signal-to-noise ratios. As a variation, the signal can also be related to the sum of signal and noise to yield a proportion of explained variance by the signal (η^*2*^). In general, every procedure augmenting signal-to-noise ratio will also increase precision (unless it also strongly increases sequence effects).

#### Reliability

In classical test theory, the *reliability* index describes the proportion of total variance of the data that results from variations of the true individual values (“true” variance between subjects divided by the total observed variance). It is conceptualized as the amount of covariation between repeated measures of the same variable (operationalized as, e.g., an odd/even split of trials; for more details see Zorowitz and Niv, 2022). Hence, it describes the ability to access between-subjects variance and relates it to the sum of between- and within-subjects variation (signal-to-noise ratio). Reliability regards the between-subjects variance as signal and (random-effect) within-subjects variance as error variance (see Figure 2). Thus, precision quantifies the amount of error variance, while reliability relates the (lack of) error variance to the total variation.

#### Accuracy

*Inaccuracy* is the difference between measured and true values of a manifest variable. Thus, while precision reflects random error of a measurement, accuracy denotes systematic deviations in manifest space (see Figure 1B). In this regard, accuracy is similar to reliability since noise is the deviation of the measurement from the true value of a manifest variable (Brandmaier et al., 2018). Accuracy can rarely be quantified since true values are usually unknown (see section on Eye-Tracking for an exception).

#### Validity

*Validity* describes the degree of conformity between manifest subject-level aggregates and either the corresponding theoretical values of the latent construct (i.e., construct validity; Figure 1A) or manifest subject-level estimates of an external criterion (i.e., criterion validity). Hence, similar to reliability, validity targets between-subjects variance and relates it to the total variation.
