# Supplementary material for: Enhancing precision in human neuroscience
Source: eLife. 2023 Aug 9;12:e85980. doi: 10.7554/eLife.85980 (PMC10411974; doi:10.7554/eLife.85980)
Supplement: Supplementary file 1. [file elife-85980-supp1.docx]

| **Theme** | **subtopic** | **notes** | **link/reference** |
| --- | --- | --- | --- |
| **General (not measurement specific)** | | | |
| Data management | BIDS |  | <https://github.com/bids-standard/bids-examples> |
| **fMRI/MRI** | | | |
| General introduction | Introductory Resources | Brief but very comprehensive book for a fast introduction into fMRI research, from recording to analyzing | Poldrack, R. A., Mumford, J. A., & Nichols, T. E. (2011). *Handbook of functional MRI data analysis*. Cambridge University Press. <https://doi.org/10.1017/CBO9780511895029> |
|  |  | Papers presenting limits and opportunities of fMRI research | Logothetis, N. K. (2008). What we can do and what we cannot do with fMRI. *Nature, 453*(7197), 869-878. <https://doi.org/10.1038/nature06976>  Heeger, D. J., & Ress, D. (2002). What does fMRI tell us about neuronal activity? *Nature reviews neuroscience, 3*(2), 142-151. <https://doi.org/10.1038/nrn730>  Geuter, S., Lindquist, M. A. & Wager, T. D. (2017). Fundamentals of Functional Neuroimaging. In J. T. Cacioppo, L. G. Tassinary, & G. G. Berntson (Eds.) *Handbook of Psychophysiology* (4th ed., pp. 41-73). Cambridge University Press. <https://doi.org/10.1017/9781107415782.004> |
| Recording guidelines | Guidelines | Fundamental paper reporting state-of-the art guidelines for fMRI data acquisition | Poldrack, R. A., Mumford, J. A., & Nichols, T. E. (2011). *Handbook of functional MRI data analysis*. Cambridge University Press. <https://doi.org/10.1017/CBO9780511895029> |
|  |  | Book presenting recommendations for fMRI recording including exemplary fMRI protocols | Filippi, M. (2016). *fMRI techniques and protocols* (2nd ed.). Humana press.  <https://doi.org/10.1007/978-1-4939-5611-1> |
| Processing guidelines | Preprocessing pipeline | Standardization of preprocessing to reduce researcher degrees of freedom and increase reproducibility | Esteban, O., Markiewicz, C. J., Blair, R. W., Moodie, C. A., Isik, A. I., Erramuzpe, A., Kent, J. D., Goncalves, M., DuPre, E., Snyder, M., Oya, H., Ghosh, S. S., Wright, J., Durnez, J., Poldrack, R. A., & Gorgolewski, K. J. (2019). fMRIPrep: A robust preprocessing pipeline for functional MRI. *Nature Methods, 16*(1), 111. <https://doi.org/10.1038/s41592-018-0235-4>  Nichols, T. E., Das, S., Eickhoff, S. B., Evans, A. C., Glatard, T., Hanke, M., Kriegeskorte, N., Milham, M. P., Poldrack, R. A., Poline, J.-B., Proal, E., Thirion, B., Van Essen, D. C., White, T., & Yeo, B. T. T. (2017). Best practices in data analysis and sharing in neuroimaging using MRI. *Nature Neuroscience, 20*(3), Article 3. <https://doi.org/10.1038/nn.4500> |
| Controlling for error variance | Toolbox | Model physiological noise to reduce error variance | Kasper, L., Bollmann, S., Diaconescu, A. O., Hutton, C., Heinzle, J., Iglesias, S., Hauser, T. U., Sebold, M., Manjaly, Z.-M., Pruessmann, K. P., & Stephan, K. E. (2017). The PhysIO Toolbox for Modeling Physiological Noise in fMRI Data. *Journal of Neuroscience Methods, 276*, 56–72. <https://doi.org/10.1016/j.jneumeth.2016.10.019> |
| Design efficiency | Toolbox | Optimize design efficiency and consequently power | <http://www.neuropowertools.org/design/start/> |
| Quality control | Toolbox | Extracts image quality metrics from structural and functional MRI data | <https://github.com/nipreps/mriqc>  Esteban, O., Birman, D., Schaer, M., Koyejo, O. O., Poldrack, R. A., & Gorgolewski, K. J. (2017). MRIQC: Advancing the automatic prediction of image quality in MRI from unseen sites. *PLOS ONE, 12*(9), e0184661. <https://doi.org/10.1371/journal.pone.0184661> |
| Power calculations | Toolbox | Power assessment based on fMRI pilot data | <http://www.neuropowertools.org/neuropower/neuropowerstart/> |
| Reporting guidelines | Guidelines | Reporting guidelines for fMRI | Poldrack, R. A., Fletcher, P. C., Henson, R. N., Worsley, K. J., Brett, M., & Nichols, T. E. (2008). Guidelines for reporting an fMRI study. *Neuroimage*, *40*(2), 409-414  <https://doi.org/10.1016/j.neuroimage.2007.11.048>  Nichols, T. E., Das, S., Eickhoff, S. B., Evans, A. C., Glatard, T., Hanke, M., ... & Yeo, B. T. (2017). Best practices in data analysis and sharing in neuroimaging using MRI. *Nature neuroscience*, *20*(3), 299-303. <https://doi.org/10.1038/nn.4500> |
|  | Pre-registration template | A template including all information necessary to pre-register an fMRI study | Beyer, F., Flannery, J., Gau, R., Janssen, L., Schaare, L., Hartmann, H., Nilsonne, G., Martin, S., Khalil, A., Lipp, I., Puhlmann, L., Heinrichs, H., Mohamed, A., Herholz, P., Sicorello, M., & Panagoulas, E. (2021). A fMRI pre-registration template. *PsychArchives*. <https://doi.org/10.23668/PSYCHARCHIVES.5121> |
| **EEG/MEG** | | | |
| General Introduction | Introductory resources | M/EEG primer | Hari, R. & Puce, A. (2017). *MEG-EEG Primer*. Oxford University Press. <https://doi.org/10.1093/med/9780190497774.001.0001>  Luck, S. J. & Kappenmann, E. S. (2017). Electroencephalography and Event-Related Brain Potentials. In J. T. Cacioppo, L. G. Tassinary, & G. G. Berntson (Eds.) *Handbook of Psychophysiology* (4th ed., pp. 74-100). Cambridge University Press.  <https://doi.org/10.1017/9781107415782.005>  Stern, R. M., Ray, W. J., & Quigley, K. S. (2000). Brain: Electroencephalography and Imaging. In R. M. Stern, W. J. Ray, & K. S. Quigley (Eds.) *Psychophysiological recording* (pp. 79-105). Oxford University Press.  <https://doi.org/10.1093/acprof:oso/9780195113594.003.0007>  Spapé, M. (2021). *A Psychologist’s guide to EEG: The electric study of the mind*. Sage. |
|  |  | General introduction for ERP technique | Luck, S.J. (2014). *An Introduction to the Event-Related Potential Technique*. The MIT Press. |
|  |  | General introduction to MEG | Hansen, P., Kringelbach, M., & Salmelin R. (2010). *MEG: An Introduction to Methods*. Oxford University Press. <https://doi.org/10.1093/acprof:oso/9780195307238.001.0001> |
|  |  | Online resource on bioelectromagnetism | Malmivuo, J. & Plonsey, R. (1995). *Bioelectromagnetism: Principles and Applications of Bioelectric and Biomagnetic Fields*. Oxford University Press. https://doi.org/10.1093/acprof:oso/9780195058239.001.0001 |
|  |  | Specific methodological considerations for the study of several ERP components | Kappenmann, E. S. & Luck, S. J. (2011). *The Oxford Handbook of Event-Related Potential Components*. Oxford University Press. <https://doi.org/10.1093/oxfordhb/9780195374148.001.0001> |
|  |  | General recommendations | Pernet, C., Garrido, M. I., Gramfort, A., Maurits, N., Michel, C. M., Pang, E., ... & Puce, A. (2020). Issues and recommendations from the OHBM COBIDAS MEEG committee for reproducible EEG and MEG research. *Nature Neuroscience*, 23(12), 1473-1483.  <https://doi.org/10.1038/s41593-020-00709-0> |
| All aspects of scientific practice | Article collection | Special Issue providing a collection of articles on methodological advances in MEG and EEG research across all aspects of scientific practice | [**https://www.sciencedirect.com/journal/neuroimage/special-issue/10RZG0FQ77G**](https://www.sciencedirect.com/journal/neuroimage/special-issue/10RZG0FQ77G) |
| Design | Toolbox | Information on computing sample size | <https://www.fieldtriptoolbox.org/example/samplesize/> |
|  |  | Recording standards | Picton, T. w., Bentin, S., Berg, P., Donchin, E., Hillyard, S. a., Johnson JR., R., Miller, G. a., Ritter, W., Ruchkin, D. s., Rugg, M. d., & Taylor, M. j. (2000). Guidelines for using human event-related potentials to study cognition: Recording standards and publication criteria. *Psychophysiology, 37*(2), 127–152. <https://doi.org/10.1111/1469-8986.3720127> |
|  |  | Guidelines for the recording and quantitative analysis of EEG data | Pivik, R. T., Broughton, R. J., Coppola, R., Davidson, R. J., Fox, N., & Nuwer, M. R. (1993). Guidelines for the recording and quantitative analysis of electroencephalographic activity in research contexts. *Psychophysiology, 30*(6), 547-558. <https://doi.org/10.1111/j.1469-8986.1993.tb02081.x> |
| Preprocessing guidelines | Tutorial videos and texts | Tutorials for data preprocessing | <https://eeglab.org/tutorials/>  <https://www.fieldtriptoolbox.org/tutorial/>  <https://mne.tools/1.1/auto_tutorials/index.html>  <https://neuroimage.usc.edu/brainstorm/Introduction>  <https://imaging.mrc-cbu.cam.ac.uk/meg/SpmAnalysis>  <https://zzz.bwh.harvard.edu/luna/tut/tut1/> |
|  |  | Pitfalls in filtering MEEG time series. The relevant literature is also covered on this page. | <https://sapienlabs.org/lab-talk/pitfalls-of-filtering-the-eeg-signal/> |
|  |  | Tutorials showcasing the decomposition of frequency domain data into periodic and aperiodic components | <https://github.com/jkosciessa/eBOSC>  <https://www.fieldtriptoolbox.org/example/irasa/>  <https://fooof-tools.github.io/fooof/> |
|  |  | Roadmap for the optimization and standardization of ERP processing and reduction pipelines | Clayson, P. E., Baldwin, S. A., Rocha, H. A., & Larson, M. J. (2021). The data-processing multiverse of event-related potentials (ERPs): A roadmap for the optimization and standardization of ERP processing and reduction pipelines. *NeuroImage, 245*, 118712. <https://doi.org/10.1016/j.neuroimage.2021.118712> |
| Controlling for error variance | Paper with Matlab scripts | Using LMM for ERP analysis (allows for repeated measures covariates) | Frömer, R., Maier, M., & Abdel Rahman, R. (2018). Group-Level EEG-Processing Pipeline for Flexible Single Trial-Based Analyses Including Linear Mixed Models. *Frontiers in Neuroscience, 12*. <https://www.frontiersin.org/articles/10.3389/fnins.2018.00048> |
|  | Paper | Baseline correction of ERP using GLM | Alday, P. M. (2019). How much baseline correction do we need in ERP research? Extended GLM model can replace baseline correction while lifting its limits. *Psychophysiology, 56*(12), e13451. <https://doi.org/10.1111/psyp.13451> |
|  | Paper/Toolbox | Hierarchical models for ERP | Pernet, C. R., Chauveau, N., Gaspar, C., & Rousselet, G. A. (2011). LIMO EEG: A Toolbox for Hierarchical LInear MOdeling of ElectroEncephaloGraphic Data. *Computational Intelligence and Neuroscience*, 2011, e831409. <https://doi.org/10.1155/2011/831409> |
|  | Paper | Periodic and aperiodic components of frequency analyses | Donoghue, T., Haller, M., Peterson, E. J., Varma, P., Sebastian, P., Gao, R., Noto, T., Lara, A. H., Wallis, J. D., Knight, R. T., Shestyuk, A., & Voytek, B. (2020). Parameterizing neural power spectra into periodic and aperiodic components. *Nature Neuroscience, 23*(12), Article 12. <https://doi.org/10.1038/s41593-020-00744-x> |
| Design efficiency | Introductory material | Design considerations for ERP studies | Luck, S.J. (2014). The Design of ERP Experiments. In *An Introduction to the Event-Related Potential Technique* (2nd ed.). The MIT Press. |
| Quality control | Quality control | Ressources from the ENIGMA consortium, e.g., analysis pipelines | <https://enigma.ini.usc.edu/ongoing/enigma-eeg-working-group/> |
|  |  | MEG recommendations and caveats | Gross, J., Baillet, S., Barnes, G. R., Henson, R. N., Hillebrand, A., Jensen, O., Jerbi, K., Litvak, V., Maess, B., Oostenveld, R., Parkkonen, L., Taylor, J. R., van Wassenhove, V., Wibral, M., & Schoffelen, J.-M. (2013). Good practice for conducting and reporting MEG research. *NeuroImage, 65*, 349–363. <https://doi.org/10.1016/j.neuroimage.2012.10.001> |
|  |  | EEG - Fully automatic preprocessing tool eliminating researchers DOF during preprocessing of single participants EEG data | <https://github.com/methlabUZH/automagic> |
|  |  | MEG - A pipeline tailored to MEG data fostering reproducible research | <https://neuosc.com/flux/>  <https://github.com/Neuronal-Oscillations/FLUX/tree/main/MNEPython> |
| Power calculations |  | Research paper explicit about power in MEG and EEG | Chaumon, M., Puce, A., & George, N. (2021). Statistical power: Implications for planning MEG studies. *NeuroImage, 233*, 117894. <https://doi.org/10.1016/j.neuroimage.2021.117894> |
| Reporting guidelines |  | Best practices in psychophysiology applying to both MEG and EEG | Gross, J., Baillet, S., Barnes, G. R., Henson, R. N., Hillebrand, A., Jensen, O., Jerbi, K., Litvak, V., Maess, B., Oostenveld, R., Parkkonen, L., Taylor, J. R., van Wassenhove, V., Wibral, M., & Schoffelen, J.-M. (2013). Good practice for conducting and reporting MEG research. *NeuroImage, 65*, 349–363. <https://doi.org/10.1016/j.neuroimage.2012.10.001>  Keil, A., Bernat, E. M., Cohen, M. X., Ding, M., Fabiani, M., Gratton, G., Kappenman, E. S., Maris, E., Mathewson, K. E., Ward, R. T., & Weisz, N. (2022). Recommendations and publication guidelines for studies using frequency domain and time-frequency domain analyses of neural time series. *Psychophysiology, 59*(5), e14052. <https://doi.org/10.1111/psyp.14052>  Styles, Suzy J., Vanja Ković, Han Ke, and Anđela Šoškić. "Towards ARTEM-IS: Design guidelines for evidence-based EEG methodology reporting tools." *NeuroImage* 245 (2021): 118721.  <https://doi.org/10.1016/j.neuroimage.2021.118721> |
|  |  | Online tool to support reporting | Šoškić, A., Kovic, V., Algermissen, J., Fischer, N. L., Ganis, G., Gau, R., … Styles, S. J. (2023, January 5). ARTEM-IS for ERP: Agreed Reporting Template for EEG Methodology - International Standard for documenting studies on Event-Related Potentials. *PsyArXiv.* <https://doi.org/10.31234/osf.io/mq5sy> |
| **EDA** | | | |
| General introduction | Textbook | Introductory textbooks | Bach, D. R. (2014). Sympathetic nerve activity can be estimated from skin conductance responses—A comment on Henderson et al. (2012). *NeuroImage, 84*, 122–123. <https://doi.org/10.1016/j.neuroimage.2013.08.030>  Dawson, M. E., Schell, A. M., & Filion, D. L. (2017). The Electrodermal System. In In J. T. Cacioppo, L. G. Tassinary, & G. G. Berntson (Eds.) *Handbook of Psychophysiology* (4th ed., pp. 217-243). Cambridge University Press.  <https://doi.org/10.1017/9781107415782.010>  Venables, P. H., & Christie, M. J. (1980). Electrodermal activity. In I. Martin & P. H. Venables (Eds.), *Techniques in psychophysiology* (pp. 3–67). John Wiley & Sons, Ltd.  Stern, R. M., Ray, W. J., & Quigley, K. S. (2000). Skin: Electrodermal Activity. In R. M. Stern, W. J. Ray, & K. S. Quigley (Eds.) *Psychophysiological recording* (pp. 79-105). Oxford University Press. <https://doi.org/10.1093/acprof:oso/9780195113594.003.0013> |
| General introduction recording guidelines, reporting guidelines, Controlling for error variance | Recommendations | Recommendations for publishing data on EDA | <https://doi.org/10.1111/j.1469-8986.2012.01384.x>  Society for Psychophysiological Research Ad Hoc Committee on Electrodermal Measures. (2012). Publication recommendations for electrodermal measurements. *Psychophysiology, 49*(8), 1017–1034. <https://doi.org/10.1111/j.1469-8986.2012.01384.x> |
| Recording guidelines, processing guidelines, design efficiency, quality control | Recommendations | Expert committee consensus guidelines | Society for Psychophysiological Research Ad Hoc Committee on Electrodermal Measures. (2012). Publication recommendations for electrodermal measurements. *Psychophysiology, 49*(8), 1017–1034. <https://doi.org/10.1111/j.1469-8986.2012.01384.x> |
| Power calculations | - | - | - |
| Reporting guidelines | Publication recommendations | Expert committee consensus guidelines | Society for Psychophysiological Research Ad Hoc Committee on Electrodermal Measures. (2012). Publication recommendations for electrodermal measurements. *Psychophysiology, 49*(8), 1017–1034. <https://doi.org/10.1111/j.1469-8986.2012.01384.x> |
|  | Data management and sharing recommendations | Data management and sharing recommendations focusing on physiological data | Ehlers, M. R., & Lonsdorf, T. B. (2022). Data sharing in experimental fear and anxiety research: From challenges to a dynamically growing database in 10 simple steps. *Neuroscience & Biobehavioral Reviews, 143*, 104958. <https://doi.org/10.1016/j.neubiorev.2022.104958> |
| **Eyetracking** |  |  |  |
| General Introduction | Blog post and teaching material | Introduction to the topic of eye- tracking and useful teaching material | <https://pupil-labs.com/blog/news/what-is-eye-tracking/> |
|  | Textbook | Introductory textbook | Holmqvist, K., Nyström, M., Andersson, R., Dewhurst, R., Jarodzka, H., & van de Weijer, J. (2011). *Eye Tracking: A comprehensive guide to methods and measures*. Oxford University Press.  Liversedge, S. P., Gilchrist, I., & Everling, S. (Eds.). (2011). *The Oxford Handbook of Eye Movements.* Oxford University Press.  <https://doi.org/10.1093/oxfordhb/9780199539789.001.0001>  Stern, R. M., Ray, W. J., & Quigley, K. S. (2000). Eyes: Pupillography and Electrooculography. In R. M. Stern, W. J. Ray, & K. S. Quigley (Eds.) *Psychophysiological recording* (pp. 79-105). Oxford University Press. <https://doi.org/10.1093/acprof:oso/9780195113594.003.0009> |
| Recording guidelines | Recommendations | Paper on how to improve data quality in eye-tracking research | Holmqvist, K., Nyström, M., & Mulvey, F. (2012). Eye tracker data quality: What it is and how to measure it. *Proceedings of the Symposium on Eye Tracking Research and Applications*, 45–52. <https://doi.org/10.1145/2168556.2168563> |
| Processing guidelines | Illustration of the effect of different fixation algorithms | Papers on methodological specifications and algorithms for the segmentation of fixations and saccades | Hessels, R. S., Niehorster, D. C., Nyström, M., Andersson, R., & Hooge, I. T. C. (2018). Is the eye-movement field confused about fixations and saccades? A survey among 124 researchers. *Royal Society Open Science, 5*(8), 180502. <https://doi.org/10.1098/rsos.180502>  Salvucci, D. D., & Goldberg, J. H. (2000). Identifying fixations and saccades in eye-tracking protocols. *Proceedings of the 2000 Symposium on Eye Tracking Research & Applications*, 71–78. <https://doi.org/10.1145/355017.355028>  Shic, F., Scassellati, B., & Chawarska, K. (2008). The incomplete fixation measure. *Proceedings of the 2008 Symposium on Eye Tracking Research & Applications*, 111–114. <https://doi.org/10.1145/1344471.1344500> |
| Controlling for error variance | Textbook |  | Holmqvist, K., Nyström, M., Andersson, R., Dewhurst, R., Jarodzka, H., & van de Weijer, J. (2011). *Eye Tracking: A comprehensive guide to methods and measures*. Oxford University Press. |
| Design efficiency | Recommendations and illustrated effects on outcome | Paper on the threats to validity in eye-tracking research | Orquin, J. L., & Holmqvist, K. (2018). Threats to the validity of eye-movement research in psychology. *Behavior Research Methods, 50*(4), 1645–1656. <https://doi.org/10.3758/s13428-017-0998-z> |
|  | Classification errors in eye-tracking research | Paper on the false positive in eye-tracking research studying reading | von der Malsburg, T., & Angele, B. (2017). False positives and other statistical errors in standard analyses of eye movements in reading. *Journal of Memory and Language, 94*, 119–133. <https://doi.org/10.1016/j.jml.2016.10.003> |
| Quality control | Test battery for eye-tracker Evaluation | This paper provides a test battery with different tasks for the evaluation of new eye-tracker models | Ehinger, B. V., Groß, K., Ibs, I., & König, P. (2019). A new comprehensive eye-tracking test battery concurrently evaluating the Pupil Labs glasses and the EyeLink 1000. *PeerJ, 7*, e7086. <https://doi.org/10.7717/peerj.7086> |
| Power calculations | - | - | - |
| Reporting guidelines | Recording and processing guidelines | Guidelines for minimal reporting in eye-tracking research | Holmqvist, K., Örbom, S. L., Hooge, I. T. C., Niehorster, D. C., Alexander, R. G., Andersson, R., Benjamins, J. S., Blignaut, P., Brouwer, A.-M., Chuang, L. L., Dalrymple, K. A., Drieghe, D., Dunn, M. J., Ettinger, U., Fiedler, S., Foulsham, T., van der Geest, J. N., Hansen, D. W., Hutton, S. B., … Hessels, R. S. (2022). Eye tracking: Empirical foundations for a minimal reporting guideline. *Behavior Research Methods*. <https://doi.org/10.3758/s13428-021-01762-8> |
| **Endocrinology** (focusing on cortisol and oxytocin as examples) | | | |
| General introduction, Recording guidelines | Tutorial paper | Tutorial for setting up everyday life studies focusing on the assessment of salivary cortisol; useful to avoid inconsistencies in study planning, data assessment and data processing | Stoffel, M., Neubauer, A. B., & Ditzen, B. (2021). How to assess and interpret everyday life salivary cortisol measures: A tutorial on practical and statistical considerations. *Psychoneuroendocrinology, 133*, 105391. <https://doi.org/10.1016/j.psyneuen.2021.105391> |
| General introduction, Recording guidelines, Reporting guidelines | Consensus guidelines | Consensus guidelines on central aspects of the assessment of the cortisol awakening response (CAR), including objective control of sampling accuracy/adherence, participant instructions, covariate accounting, sampling protocols, quantification strategies as well as reporting and interpreting of CAR data | Stalder, T., Kirschbaum, C., Kudielka, B. M., Adam, E. K., Pruessner, J. C., Wüst, S., Dockray, S., Smyth, N., Evans, P., Hellhammer, D. H., Miller, R., Wetherell, M. A., Lupien, S. J., & Clow, A. (2016). Assessment of the cortisol awakening response: Expert consensus guidelines. *Psychoneuroendocrinology, 63*, 414–432. <https://doi.org/10.1016/j.psyneuen.2015.10.010> |
| General introduction, Recording guidelines, Reporting guidelines | Consensus guidelines | Critical evaluation and update of current cortisol awakening response (CAR) consensus guidelines including recent technological advances | Stalder, T., Lupien, S. J., Kudielka, B. M., Adam, E. K., Pruessner, J. C., Wüst, S., Dockray, S., Smyth, N., Evans, P., Kirschbaum, C., Miller, R., Wetherell, M. A., Finke, J. B., Klucken, T., & Clow, A. (2022). Evaluation and update of the expert consensus guidelines for the assessment of the cortisol awakening response (CAR). *Psychoneuroendocrinology, 146*, 105946. <https://doi.org/10.1016/j.psyneuen.2022.105946> |
| Processing guidelines, Quality control | Protocol specifications | Specific protocols and biochemical reagents are given from manufacturers including control samples to check quality | (consult the manufacturer’s manuals) |
| General introduction, Recording guidelines, Reporting guidelines | Recommendations and tools for data collection and data management | Menstrual cycle studies: appropriate study design and sampling strategy, managing demand characteristics, measuring menstrual bleeding dates, ovarian hormones, and ovulation; suggestions for data preparation and coding of menstrual cycle day and phases, data visualization, statistical modeling | Schmalenberger, K. M., Tauseef, H. A., Barone, J. C., Owens, S. A., Lieberman, L., Jarczok, M. N., Girdler, S. S., Kiesner, J., Ditzen, B., & Eisenlohr-Moul, T. A. (2021). How to study the menstrual cycle: Practical tools and recommendations. *Psychoneuroendocrinology, 123*, 104895. <https://doi.org/10.1016/j.psyneuen.2020.104895> |
| General introduction, Recording guidelines, Reporting guidelines, Quality control | Tutorial,  recommendations | Recommendations for the standardization of intransal oxytocin administration, guidelines for reporting on intransal oxytocin administration | Guastella, A. J., Hickie, I. B., McGuinness, M. M., Otis, M., Woods, E. A., Disinger, H. M., Chan, H.-K., Chen, T. F., & Banati, R. B. (2013). Recommendations for the standardisation of oxytocin nasal administration and guidelines for its reporting in human research. *Psychoneuroendocrinology, 38*(5), 612–625. <https://doi.org/10.1016/j.psyneuen.2012.11.019> |
| General introduction, Recording guidelines,  Processing guidelines, Reporting guidelines, Data management guidelines, Quality control, Controlling for error variance | Tutorial, recommendations | Recommendations for the standardization of peripheral oxytocin measurement; guidelines for reporting on peripheral oxytocin measurement | Tabak, B. A., Leng, G., Szeto, A., Parker, K. J., Verbalis, J. G., Ziegler, T. E., Lee, M. R., Neumann, I. D., & Mendez, A. J. (2023). Advances in human oxytocin measurement: Challenges and proposed solutions. *Molecular Psychiatry, 28*(1), Article 1. <https://doi.org/10.1038/s41380-022-01719-z> |
| General introduction, Design efficiency, Power calculations | Recommendations | Recommendations for improving methodological standards for intranasal oxytocin research | Quintana, D. S., Lischke, A., Grace, S., Scheele, D., Ma, Y., & Becker, B. (2021). Advances in the field of intranasal oxytocin research: Lessons learned and future directions for clinical research. *Molecular Psychiatry, 26*(1), Article 1. <https://doi.org/10.1038/s41380-020-00864-7> |
| Controlling for error variance | Recommendations | Recommendations for covariates to be considered in (tonic and phasic) cortisol measurements | Strahler, J., Skoluda, N., Kappert, M. B., & Nater, U. M. (2017). Simultaneous measurement of salivary cortisol and alpha-amylase: Application and recommendations. *Neuroscience & Biobehavioral Reviews, 83*, 657–677. <https://doi.org/10.1016/j.neubiorev.2017.08.015> |
| Controlling for error variance | Recommendations | Potential covariates of endogenous oxytocin levels based on meta-analysis that can inform study design decisions | Engel, S., Laufer, S., Miller, R., Niemeyer, H., Knaevelsrud, C., & Schumacher, S. (2019). Demographic, sampling- and assay-related confounders of endogenous oxytocin concentrations: A systematic review and meta-analysis. *Frontiers in Neuroendocrinology, 54*, 100775. <https://doi.org/10.1016/j.yfrne.2019.100775> |
| Quality control | Checklist to evaluate quality of studies reporting cortisol data | The Cortisol Assessment List (CoAL) A tool to systematically document and evaluate cortisol assessment in blood, urine and saliva | Laufer, S., Engel, S., Lupien, S., Knaevelsrud, C., & Schumacher, S. (2022). The Cortisol Assessment List (CoAL) A tool to systematically document and evaluate cortisol assessment in blood, urine and saliva. *Comprehensive Psychoneuroendocrinology, 9*, 100108. <https://doi.org/10.1016/j.cpnec.2021.100108> |
| Reporting guidelines | Field-specific guidelines for open and reproducible science | Opportunities presented by the application of open and reproducible scientific practices in psychoneuroendocrinology; introduction to the topics preregistration, registered reports, quantifying the impact of equally-well justifiable analysis decisions, and open data and scripts; concrete steps for future actions and links to additional resources | Meier, M., Lonsdorf, T. B., Lupien, S. J., Stalder, T., Laufer, S., Sicorello, M., Linz, R., & Puhlmann, L. M. C. (2022). Open and reproducible science practices in psychoneuroendocrinology: Opportunities to foster scientific progress. *Comprehensive Psychoneuroendocrinology, 11*, 100144. <https://doi.org/10.1016/j.cpnec.2022.100144> |
